# Comparative Analysis of Genomic Differentiation and Outcomes of Contact Between Taxa Within a Species Complex of North Temperate Fishes

**DOI:** 10.1002/ece3.73685

**Published:** 2026-07-21

**Authors:** E. B. Taylor, A. Geraldes

**Affiliations:** ^1^ Department of Zoology, Biodiversity Research Centre and Beaty Biodiversity Museum University of British Columbia Vancouver British Columbia Canada; ^2^ Departments of Zoology and Botany, Biodiversity Research Centre University of British Columbia Vancouver British Columbia Canada

## Abstract

The outcomes of contact between closely‐related species provide insights into the processes that influence divergence along the speciation continuum. We conducted a comparative genomic analysis of divergence between several taxa of *Salvelinus* fishes from the North Pacific that vary in estimated divergence times from 0.8 to 5.2 million years ago. We found a general increase in the extent of reproductive isolation, as assessed by the presence and kinds of hybrids produced in sympatry, and estimated divergence time. There was evidence of less effective gene flow between *Salvelinus confluentus* (bull trout) and *S. malma lordi* (Southern Dolly Varden) in sympatry than between the more recently diverged *S. alpinus* (Arctic char) and *S. m. malma* (Northern Dolly Varden) in sympatry. By contrast, although *S. m. malma* and *S. m. lordi* are estimated to have diverged from each other only slightly more recently than *S. alpinus* and *S. malma* (1.3–1.4 million years ago, respectively), the former produced hybrid swarms upon contact in two lakes while the latter pair showed pronounced, but incomplete, reproductive isolation while sympatric in three lakes. Genome wide assessments of divergence between pairs of taxa suggested that restriction of gene flow between each pair is widespread across the genome, consistent with advanced reproductive isolation between *S. confluentus* and *S. m. lordi* and *S. alpinus* and *S. m. malma,* but contrasts with an apparent lack of reproductive isolation between *S. m. malma* and *S. m. lordi.* We suggest that differences in reproductive ecology (or lack thereof) between pairs of taxa in sympatry likely contribute to variation in their position along the speciation continuum.

## Graphical abstract

We use comparative genomic comparisons between species and subspecies in a north temperate fish to examine the relationship between inferred divergence time and extent of reproductive isolation. We found that similar divergence times were associated with dramatically distinct levels of reproductive isolation. We suggest that ecological differences in spawning ecology may lead to distinct outcomes following secondary contact.

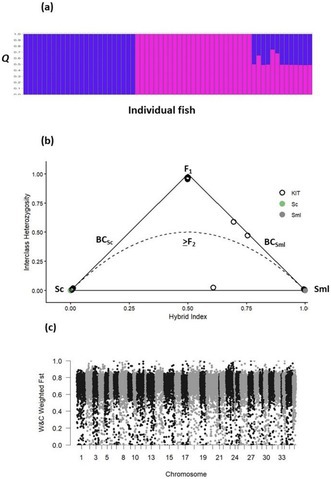

## 1 Introduction

One of the most fundamental questions in biology is how new species originate. Although the process of speciation may be represented by a continuum of divergence, groups of organisms are typically considered to be distinct species when there is no effective gene flow between them, that is, they are reproductively isolated from one another. Among myriad uncertainties concerning the process of speciation are questions such as: at what pace does reproductive isolation evolve, is reproductive isolation best described as a continuous process, or as a discrete event that results in distinct species once some threshold of divergence is reached, and what processes influence the pace and temporal mode of speciation (Coyne and Orr 1989; Stankowski and Ravinet 2021)? One key uncertainty related to such questions is whether or not time since divergence between two populations is associated with a gradual (linear or nonlinear) increase in the extent of reproductive isolation (Coyne and Orr 1989). Here, we consider reproductive isolation to mean the extent of gene flow between populations through the production of post‐*F*_1_ hybrids and backcrosses (see Westram et al. 2022). For instance, when divergence in allopatry is driven either by neutral processes or incidentally by consistent divergent natural selection, and with all else being equal, populations that have diverged over a longer time period, will in general, be further along in the speciation process than more recently diverged populations (Coyne and Orr 1989; Matute and Cooper 2021). Alternatively, if natural selection acts directly on traits that promote reproductive isolation, for example, mate choice, reproductive habitat use, traits that depress hybrid fitness, or if rapid genetic drift occurred during a founder event associated with allopatric separation, then the relationship between time since divergence and the extent of reproductive isolation may be more complex (Coyne and Orr 1989; Stankowski and Ravinet 2021).

Key systems that have been exploited to address such questions and issues are contact zones, that is, geographic areas where divergent lineages come into contact and may exchange genes to varying degrees (e.g., Johannesson et al. 2020; Wait and Peñalba 2025). Contact may result in a lack of gene flow and the maintenance of completely reproductively (and hence genetically) isolated populations, the production of a hybrid swarm from the lack of any reproductive isolation, or any intermediate condition along what has been termed the “speciation continuum” (e.g., Johannesson et al. 2020; Stankowski and Ravinet 2021). In particular, the comparative study of replicate pairs of populations and taxa still capable of exchanging genes in contact zones and that have diverged over variable time periods are especially useful and needed in these contexts (e.g., Coyne and Orr 1989; Seehausen et al. 2014; Supple et al. 2015; Chase et al. 2021; Irwin et al. 2025; Okude et al. 2026). The development of genome wide sequence analyses has provided novel approaches with unprecedented resolution to address such issues within the context of “speciation genomics” (e.g., Delmore et al. 2018; Irwin et al. 2018; Henderson and Brelsford 2020; Chase et al. 2021; Shang et al. 2023; Ravinet et al. 2017; Liu et al. 2020; Yamasaki et al. 2025).

**FIGURE 1:**
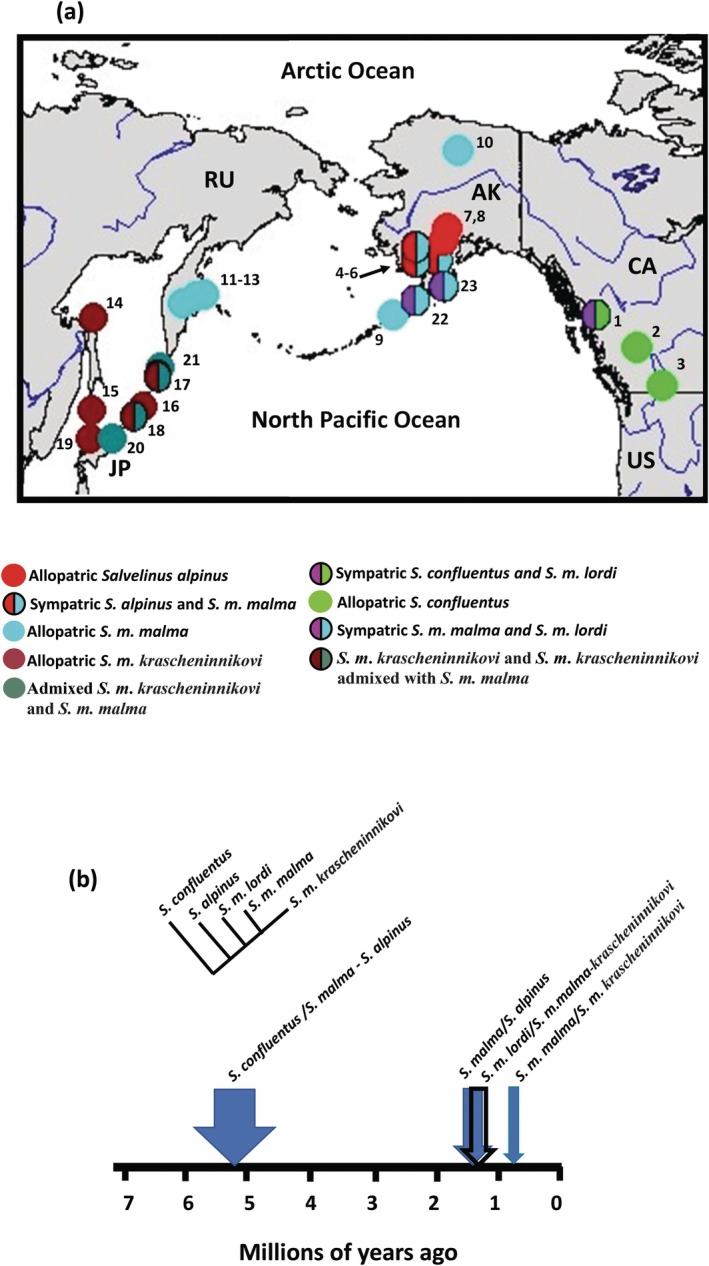
(a) Map of main sample localities for *Salvelinus confluentus*, *S. alpinus*, and *S. malma*. Red = *S. alpinus*, cyan = *S. m. malma*, purple = *S. m. lordi*, dark red = *S. m. krascheninnikovi*, light green = *S. confluentus*, dark green = *S. m. krascheninnikovi* admixed with *S. m. malma*. Localities with two colors are those with sympatric taxa and some admixture between taxa. RU = Russia, JP = Japan, US = United States, AK = Alaska, CA = Canada. Numbers accompanying symbols refer to location numbers in Table 1 and Table S1. Major rivers are shown as light blue lines. Sample localities shown are those used in Admixture analyses of sympatric taxa, as unknowns in Triangle Plot analyses, and in pairwise F_ST_ and d_xy_ analyses. Other localities used as parental populations in triangle plot analyses are given in Table S1 and depicted in Taylor et al. (2025). (b) Summary of phylogenetic relationships (upper) and divergence time estimates (millions of years ago) between taxa (lower). The point of each downward arrow is the mean of the posterior distribution of estimated divergence times, and the width of the arrow heads represents the approximate 95% credible limits. Estimates were based on a divergence with time estimate using BEAST2 (Bouckaert et al. 2014) and 9,433 single nucleotide polymorphisms (see Taylor et al. 2025). The arrow showing divergence between *S. m. lordi* and the common ancestor of *S. m. malma* and *S. m. krascheninnikovi* in black outline is displaced slightly to the right to increase its visual distinction from the arrow representing divergence between *S. alpinus* and *S. malma*.

One such group of taxa involves the three species (and their subspecies) constituting what was once known as the Arctic char ( *Salvelinus alpinus* ) complex (McPhail 1961). In the North Pacific and adjacent Arctic Ocean basins, this complex consists of three now widely‐recognized species: the Arctic char, Dolly Varden ( *S. malma* ), and bull trout ( *S. confluentus* ; see Taylor 2016; Weinstein et al. 2024). *Salvelinus alpinus* and *S. malma* are largely allopatric but do come into contact in a few lakes in northeastern Siberia, southwestern Alaska and in some streams of the Coronation Gulf region of the western Canadian Arctic (DeLacy and Morton 1943; Taylor 2016; Esin et al. 2017; Weinstein et al. 2024). In sympatry, the two species are strongly differentiated ecologically and genetically with little contemporary gene flow between them (DeLacy and Morton 1943; Gharrett et al. 1991; Taylor et al. 2008; May‐McNally et al. 2015; Dennert et al. 2016; Smith et al. 2025a, 2025b). Similarly, *S. malma* and *S. confluentus* are largely allopatric, but several sympatric populations occur along the crest of the Coastal‐Cascade mountains and adjacent areas (e.g., Baxter et al. 1997; Leary and Allendorf 1997; Taylor 2016). Here, *S. confluentus* and *S. malma* not infrequently hybridize, but maintain themselves as distinct gene pools (Redenbach and Taylor 2003). *Salvelinus alpinus* and *S. confluentus* are not currently known to coexist. Although several subspecies have been described in *S. alpinus* , the three well‐recognized subspecies of *S. malma* : *S. m. malma*, *S. m. lordi*, and *S. m. krascheninnikovi* (considered synonymous with *S. curilus* , see Weinstein et al. 2024), have been better studied in terms of how much they overlap geographically and exchange genes (Salmenkova et al. 2000; Radchenko 2004; Yamamoto et al. 2021; Liu et al. 2024; Taylor et al. 2025). In summary, the three species occur as replicate sets of taxa that may occur in sympatry: *S. alpinus* and *S. malma* ; *S. malma* and *S. confluentus* ; *S. m. malma* and *S. m. lordi* (in northwestern North America) and *S. m. krascheninnikovi* and *S. m. malma* (in northeastern Asia). Divergence times between *S. alpinus* /*malma* and *S. confluentus* , between *S. alpinus* and *S. malma* , and among subspecies of *S. malma* are estimated to range from about 5.2 million years ago (mya), 1.4 mya, to 0.8–1.3 mya, respectively (Figure 1; Taylor et al. 2025).

Here, we used genomic data gathered from species, subspecies and populations of *S. alpinus* , *S. malma* , and *S. confluentus* that are at different points along the speciation continuum and currently meet in nature to pose several questions: To what extent do the different sympatric taxa hybridize if at all? If they do hybridize, what sorts of hybrids are formed and is hybridization introgressive (i.e., do genes from one species become incorporated into the background of the other)? How does the landscape of differentiation vary between sympatric species and subspecies and along the continuum of speciation? Finally, we assessed variation in the outcome of contact between taxa along a speciation continuum to test if the extent of reproductive isolation between pairs of taxa has accumulated as a direct function of estimated time since divergence among these taxa (Taylor et al. 2025) or if the relationship is more complex.

## 2 Methods

### 2.1 Diversity of *Salvelinus* Samples

We examined 764 DNA samples from a database of 909 *Salvelinus* tissues acquired over the past 25 years used in various studies on char evolutionary ecology (see Taylor et al. 2025). Here, we focussed on samples of coexisting taxa from Alaska, Russia (including the Kuril Islands), British Columbia as well as select allopatric samples from these and adjacent areas: *S. alpinus eurythrinus, S. confluentus, S. malma malma, S. m. lordi*, and *S. m. krascheninnikovi* (Figure 1; Table 1; Table S1). Original field collections in areas of known or suspected sympatry were made using a migratory fence trap on the Kitwanga River, and beach seining, and minnow trapping over a diversity of sites at the other locations (see Taylor et al. 2008, May‐McNally et al. 2015; Liu et al. 2024; Maier 2025), with no known bias towards one taxon or the other or hybrid (admixed) individuals. Samples were assigned to presumptive taxon based on independent biogeographical, morphological, and genetic data as detailed in Taylor et al. (2025). All original fish tissue collections were made following UBC Animal Care Committee approved protocols.

**TABLE 1:** List of taxa sampled, their geographic localities, and sample sizes.

Taxon 1	Locality	*N*	Taxon 2	Locality	*N*
*Salvelinus confluentus*	1. Kitwanga River, bc 55.10–128.09	28^a^	*Salvelinus malma lordi*	Kitwanga River, bc 55.10–128.09	34^b^
*Salvelinus confluentus*	2. Quesnel Lake, bc 52.53–121.12	24	*Salvelinus confluentus*	3. Kootenay Lake, bc 49.57–116.83	2
*Salvelinus alpinus* ^c^	4. Aleknagik Lake, AK 59.33–158.83	28	*Salvelinus* * malma malma*	4. Aleknagik Lake, AK 59.33–158.83	23
*Salvelinus alpinus*	5. Iliamna Lake, AK 59.44–155.74	22	*Salvelinus m. malma*	5. Iliamna Lake, AK 59.44–155.74	53
*Salvelinus alpinus*	6. Lake Nerka, AK 59.57–158.81	30	*Salvelinus m. malma*	6. Lake Nerka, AK 59.57–158.81	31
*Salvelinus alpinus*	7. Caribou Lakes, AK 60.45–154.0	24	*Salvelinus alpinus*	8. Lower Tazimina Lake, AK 59.99–154.49	15
*Salvelinus m. malma*	9. Frosty Creek, AK 55.17–162.81	19	*Salvelinus m. malma*	10. Upper Anaktuvuk R., AK 68.14–151.53	15
*Salvelinus m. malma*	11–13. Shchapina R., RU, Kamchatka R., RU, Azabach'ye Lake, RU 55.44159.49; 56.24162.46; 56.18161.86	9	*Salvelinus m. krascheninnikovi*	14–19. Tailki R., Sakhalin Is., RU, Aniva R., Sakhalin, RU, Unnamed stream, Urup Is., Kuril Is., RU, Unnamed River, Onekotan Is., Kuril Is., RU; Unnamed stream, Simushir Is., Kuril Is., RU; Sorachi R., JP 54.29142.81; 46.70142.52; 46.22150.32; 49.62154.83; 47.10152.14; 44.36142.43	12
*Salvelinus m. malma*	10. Upper Anaktuvuk R., AK 68.14–151.53	14	*Salvelinus m. lordi*	Kit 1. Kitwanga River, bc 55.10–128.09	25
*Salvelinus m. malma* x *S. m. lordi*	22. Chignik Lake, AK 56.26–158.85	187	NA		
*Salvelinus m. malma* x *S. m. lordi*	23. Karluk Lake, AK 57.38–154.05	15	NA		
*Salvelinus m. malma* x *S. m. krascheninnikovi*	16, 17, 20, 21. Unnamed stream, Urup Is., Kuril Is., RU, Unnamed River, Onekotan Is., Kuril Is., RU; Krasheninnikova R., Paramushir Is., Kuril Is., RU; Kruglovsky Isthmus, Kunashir Is. Kuril Is., RU 46.22150.32; 49.62154.83; 50.29155.34; 44.42146.31	7	NA		

*Note:* Two samples listed on the same line represent the two taxa (or populations within taxa) that were compared for genome‐wide *F*_ST_ and *d*_xy_. Taxa underlined represent those used in Admixture analysis within the localities indicated. Two taxa listed in the same column and separated by an “*x*” represent admixed populations.

Abbreviations: AK, Alaska, US; BC, British Columbia, Canada; JP, Japan; RU, Russia.

^a^ Includes 4 fish with some degree of admixture with *S. m. lordi*.

^b^ Includes 9 fish with some degree of admixture with *S. confluentus* .

^c^ All are *S. alpinus eurythrinus* (see Taylor et al. 2025).

### 2.2 DNA Extractions and Quality Control

Total genomic DNA was extracted from tissues using the DNeasy Blood & Tissue kit and eluted in AE buffer (Qiagen Inc., Valencia, CA, USA). The concentration, purity, and integrity of DNA extracts were measured using fluorometry, spectrophotometry, and agarose gel electrophoresis as described by Taylor et al. (2025).

### 2.3 Genotyping‐By‐Sequencing

We used a reduced representation genome sequencing approach, genotyping‐by‐sequencing (GBS; Elshire et al. 2011), to generate sequence data from a representative fraction of the genome. We used a modified GBS protocol (Alcaide et al. 2014; Toews et al. 2016; Taylor et al. 2025) to generate pooled libraries of digested and individually barcoded DNA. The samples used in this study were sequenced over seven sequencing lanes between 2019 and 2023 that included many other samples from a diversity of species and projects, of which 909 samples were from the genus *Salvelinus*. The full details of preparation of samples and DNA library construction and sequencing are given in Taylor et al. (2025).

### 2.4 SNP Discovery Bioinformatics

Analysis of the sequence data (demultiplexing, trimming, reference genome alignment and genotype calling) followed a bioinformatics pipeline available at https://doi.org/10.5061/dryad.t951d (Irwin et al. 2016) for GBS read processing and mapping with modifications as fully described by Taylor et al. (2025). The “Master” variant call format (VCF) file generated contained 7,218,090 variant sites.

We filtered the Master VCF file using VCFtools v0.1.11 (Danecek et al. 2011) to remove any individuals that had more than 70% missing data; average (SD) missing data across individuals was 0.144 (0.120, Table S1). Next, we used VCFtools to remove indels and retain only biallelic SNPs with less than 70% missing data, a minimum genotype quality of 20 (‐‐min‐GQ 20), and a minor allele frequency of 0.01 (‐‐maf 0.01, *N* = 236,777 SNPs). We then eliminated SNPs that showed an observed heterozygosity of 0.6 or higher, as these are likely the result of mapping to paralogous regions of the genome, using a custom script (Owens et al. 2016; *N* = 229,712 SNPs). We then used VCFtools to create a set of VCF files containing sub‐samples of fish from the Master VCF according to a set of specific inter‐ and intra‐taxon pairwise comparisons, each consisting of a single pair of populations except where noted (Table 1). These 11 VCFs comprised (i) sympatric *S. confluentus* and *S. m. lordi*, (ii)—(iv) three Alaskan lakes each with sympatric *S. alpinus* and *S. m. malma*, (v) allopatric *S. alpinus* , (vi) allopatric *S. m. malma*, (vii) allopatric *S. m. malma* and allopatric *S. m. lordi*, (viii) allopatric *S. confluentus* , (ix) allopatric *S. m. malma*, allopatric *S. m. lordi*, and an admixed population of these taxa from the Chignik Lake watershed in southwestern Alaska, (x) allopatric *S. m. malma*, allopatric *S. m. lordi*, and an admixed population of these taxa from Karluk Lake in southwestern Alaska (see Taylor and May‐McNally 2015; Liu et al. 2024), and (xi) allopatric *S. m. malma* from Russia and allopatric *S. m*. *krascheninnikovi* from Japan (Hokkaido) and Russia (Sakhalin and Kuril Islands). In the samples of set xi, the allopatric *S. m. malma* and *S. m*. *krascheninnikovi* were from multiple localities as sample numbers for allopatric *S. m. malma* and *S. m*. *krascheninnikovi* from single Asian localities were low (< 10). Paired samples of allopatric populations within taxa ( *confluentus* , *S. alpinus*, and *S. m. malma*) were included to provide some scale of reference for comparisons between taxa.

### 2.5 Population Genomic Analyses

We used Admixture v1.3.0 (Alexander et al. 2009) to estimate the number of genetic ancestry groups (*K*) that best represented genotype data and the estimated proportional ancestry (*Q*) that each fish comprises for sympatric *S. confluentus* and *S. m. lordi* from one locality, and *S. m. malma* and *S. alpinus* in three Alaskan lakes (Table 1). We did not conduct Admixture analyses on other paired samples as they were allopatric samples (e.g., two *S. alpinus* samples from two Alaskan lakes; *S. m. malma* and *S. m. krascheninnikovi* from Asian localities), sample sizes were small (< 20), and/or previous analyses (Liu et al. 2024; Taylor et al. 2025) had already established that the localities sampled were admixed populations of two taxa (e.g., *S. m. malma* and *S. m. lordi* in Chignik and Karluk lakes, AK, and *S. m. malma* and *S. m. krascheninnikovi* from the Kuril Islands). We ran five replicates (unsupervised) for each *K* from 1 to 5 and terminated each run when the difference in likelihoods between successive iterations fell below 1 × 10^−6^. We evaluated different levels of Admixture *K's* based on that which minimized the cross validation error (CVE, Alexander et al. 2009). Here, the input paired‐sample VCF files were further filtered to remove sites in close gametic disequilibrium by using Plinkv1.9 to eliminate SNPs with *r* ^2^ > 0.2 in overlapping windows of 50 consecutive SNPs moving 10 SNPs at a time between windows (option “‐‐indep‐pairwise 50 10 0.2”) resulting in files with between 15,299 and 46,866 SNPs.

Next, we created triangle plots of interclass heterozygosity versus hybrid index using the R package *triangulaR* (Wiens et al. 2025). Here, the interclass heterozygosity of an individual is defined as the proportion of loci with differences in frequency between parental lineages of 1.0 that were heterozygous for the alternatively diagnostic parental alleles. The hybrid index of an individual is the proportion of ancestry from one parental population. Under Hardy–Weinberg Equilibrium, it is possible to predict the position of an individual based on its observed interclass heterozygosity and hybrid index over the generations following interbreeding between parental populations (Wiens et al. 2025). Sample VCFs included fish from one or more populations of both parental taxa as determined by Taylor et al. (2025). Instead of using Plink to filter the paired‐samples VCF files for sites in linkage disequilibrium as described above, we used VCFtools to include only sites that were no closer to 1 kb from each other to accommodate *triangulaR*'s use of VCF files as input (rather than binary genotype files or “bed” files, used in Admixture). This resulted in VCF files with from 44,389 to 53,110 SNPs for the paired sample files described above. Calculations of interclass heterozygosity and ancestry proportion were based on loci with SNPs that were fixed for alternative alleles for each parental population (as determined by *triangulaR*) which ranged from 203 (*S. m. malma* vs. *S. m. krascheninnikovi*) to 12,149 SNPs ( *S. confluentus* vs. *S. m. lordi*).

### 2.6 Calculation of Differentiation Across the Genome

We calculated pairwise estimates of Weir and Cockerham's *F*_ST_ (Weir and Cockerham 1984), the proportion of allele frequency variability attributable to differences between pairs of samples, across SNPs in the autosomal datasets (without removal of SNPs in close linkage) using VCFtools. Each paired‐sample VCF consisted of between 70,475 and 105,639 SNPs for the paired sample files described above. Because all the samples from the Chignik Lake watershed were admixed between *S. m. malma* and *S. m. lordi* (see Results), we placed individuals into “*S*. *m. malma*‐like” (Q_*S.m.malma*_ > 0.5) and “*S. m. lordi*‐like” (Q_*S.m.lordi*_ > 0.5) groups. Estimates of *F*_ST_ were calculated across 100 kb sliding windows of SNPs with sliding step size set to 25 kb. Estimates were weighted by the number of samples at each SNP and by the numbers of SNPs used in the calculations within each window and then averaged across windows. Weighted average values of *F*_ST_ per pairwise contrast or across chromosomes within taxa were visualized using violin and Manhattan plots in the R packages ggplot2 or qqman, respectively (Turner 2018; R Core Team 2024). Estimates of *F*_ST_ were not calculated for samples in Karluk Lake, AK, or the Kuril Islands owing to small sample sizes (< 20). Given that *F*_ST_ can be influenced by variation in effective population sizes of one or both of the samples being examined, we also calculated *d*_xy_, the absolute measure of nucleotide divergence between populations, which is not influenced by within population diversity which we assessed with *π*, within population nucleotide diversity (Nei 1987). We used pixy (Korunes and Samuk 2021) to calculate *d*_xy_ and *π* across invariant and variant sites generated using GATK4 (McKenna et al. 2010; Van der Auwera and O'Connor 2020) as described by Taylor et al. (2025), but incorporating the ‐‐all‐sites option (see Korunes and Samuk 2019). The *d*_xy_ and *π* calculations were also made across 100 kb sliding windows of SNPs with sliding step size set to 25 kb. The “all sites” MASTER VCF used in the pixy calculations contained 23,002,935 variant and invariant sites.

## 3 Results

### 3.1 Genomic Outcomes of Sympatry: *Salvelinus confluentus* and *S. malma lordi*

The number of reads mapped to the reference genome with a minimum quality of 20 ranged from 6.98 million to 7.85 million covering about 1.9 to 2.2% of the genomes of *S. alpinus* , *S. malma* , and *S. confluentus* (Table S1).

**FIGURE 2:**
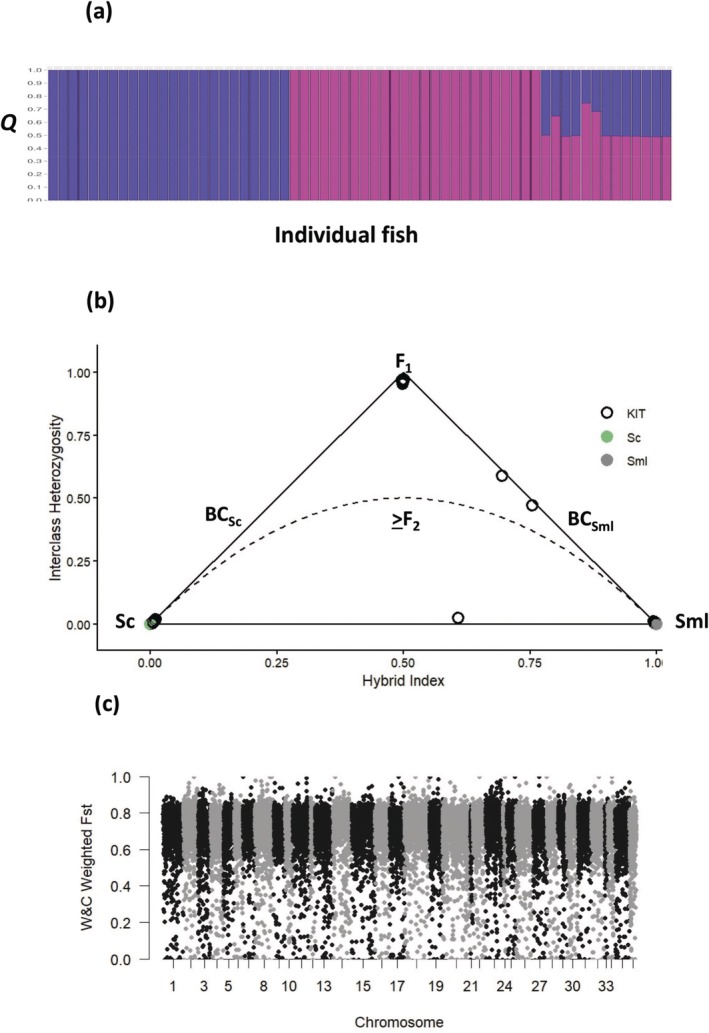
Differentiation between sympatric *Salvelinus confluentus* and *S. malma* *lordi* from the Kitwanga River, British Columbia examined by (a) unsupervised Admixture (Alexander et al. 2009), (b) Triangle Plot analysis and (c) weighted mean Weir and Cockerham's (1984) *F*_ST_. In (a), the *y*‐axis is the proportion of ancestry (*Q*) within *K* = 2 genetic groups (blue = *S. confluentus* , mauve = *S. m. lordi*) for each individual fish (thin vertical lines) inferred from 15,299 single nucleotide polymorphisms. Metrics for models for *K* = 1–5 can be found in Table A1. In (b), the Triangle Plot shows projection of interclass heterozygosity by hybrid index (0 = *S. confluentus* , 1.0 = *S. m. lordi*), estimated using unlinked loci with fixed differences between parental taxa. Sc = parental samples of *S. confluentus* (green points in lower left corner), Sml = parental samples of *S. m. lordi* (gray points in lower right corner); Kitwanga River samples are open circles. Approximate positions along the triangle or the dotted line show those expected for *S. confluentus* (Sc), *S. m. lordi* (Sml), *F*_1_ hybrids between *S. confluentus* and *S. m. lordi*, *F*_2_ and later generation hybrids (≥ *F*_2_) and backcross generation 1 to *S. confluentus* (BC_Sc_) or *S. m. lordi* (BC_Sml_). Successive backcross generations will occupy positions between BC_Sc_ and *S. confluentus* (or BC_Sml_ and *S. m. lordi*). Many points overlap; individual scores are given in Table A2. In (c) shown is a Manhattan plot of weighted mean *F*_ST_ (Weir and Cockerham 1984) across 36 autosomal chromosomes calculated across 100 kb sliding windows with a window step size of 25 kb using total of 102,110 single nucleotide polymorphisms.

**FIGURE 3:**
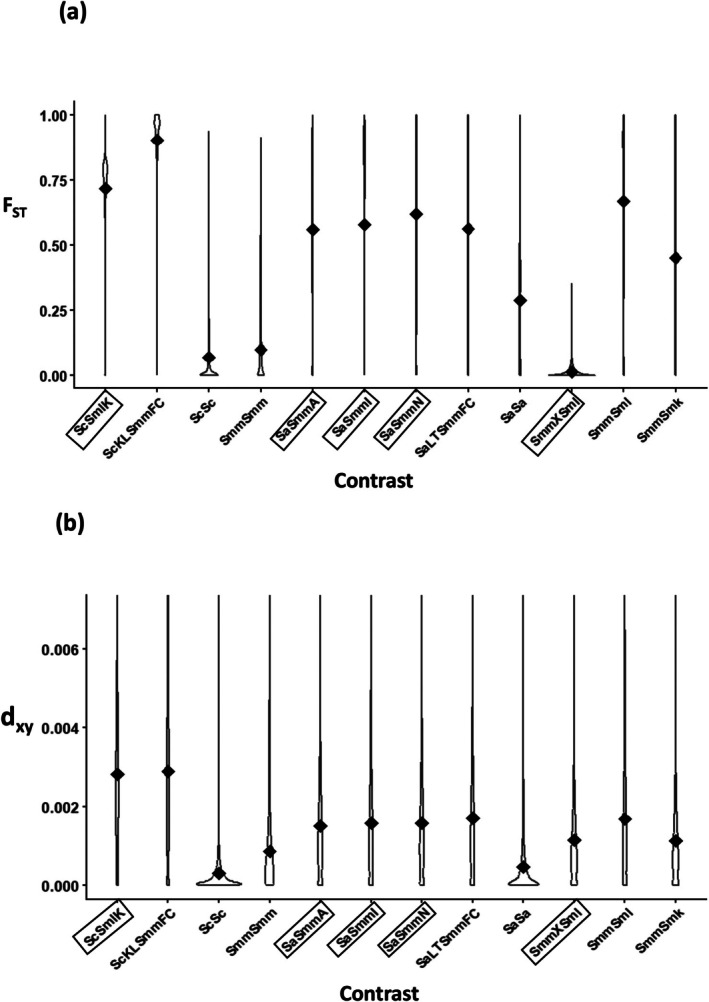
Violin plots of kernel probability distribution of (a) weighted mean Weir and Cockerham's (1984) *F*_ST_ and (b) *d*_xy_ (Nei 1987) between different sympatric (*x*‐axis labels enclosed in rectangles) and allopatric population samples of *Salvelinus confluentus, S. alpinus, S. malma malma, S. m. lordi* and *S. m. krascheninnikovi*. Estimates were calculated from the weighted means across 100 kb sliding windows (window step size = 25 kb) and then averaged across all 36 autosomal chromosomes (black diamond). Vertical lines represent range of values. ScSmlK = *S. confluentus* and *S. m. lordi* from the Kitwanga, R., British Columbia (BC); ScKLSmmFC = *S. confluentus* Kootenay Lake, BC, and *S. m. malma* from Frosty Creek, AK, ScSc = *S. confluentus* from Kootenay and Quesnel lakes, BC; SmmSmm = *S. m. malma* from an unnamed tributary of the Upper Anaktuvuk River and Frosty Creek, Alaska (AK); SaSmmA, SaSmmI, and SaSmmN = *S. alpinus* and *S. m. malma* from Lake Aleknagik, Iliamna Lake, and Lake Nerka, AK, respectively; SaLTLSmmFC = *S. alpinus* from Lower Tazimina Lake, AK, and *S. m. malma* from Frosty Creek, AK, SaSa = *S. alpinus* from Caribou Lakes and Lower Tazimina Lake, AK; SmmXSml, admixed *S. m. malma* and *S. m. lordi* from Chignik Lake, AK; SmmSml = *S. m. malma* and *S. m. lordi* from an unnamed tributary of the Upper Anaktuvuk River. AK, and the Kitwanga, River, BC (fish admixed with *S. confluentus* removed), respectively; SmmSmk = *S. m. malma* and *S. m. krascheninnikovi* from several localities in the northwestern Pacific (see Table 1, Table S1).

**FIGURE A1:**
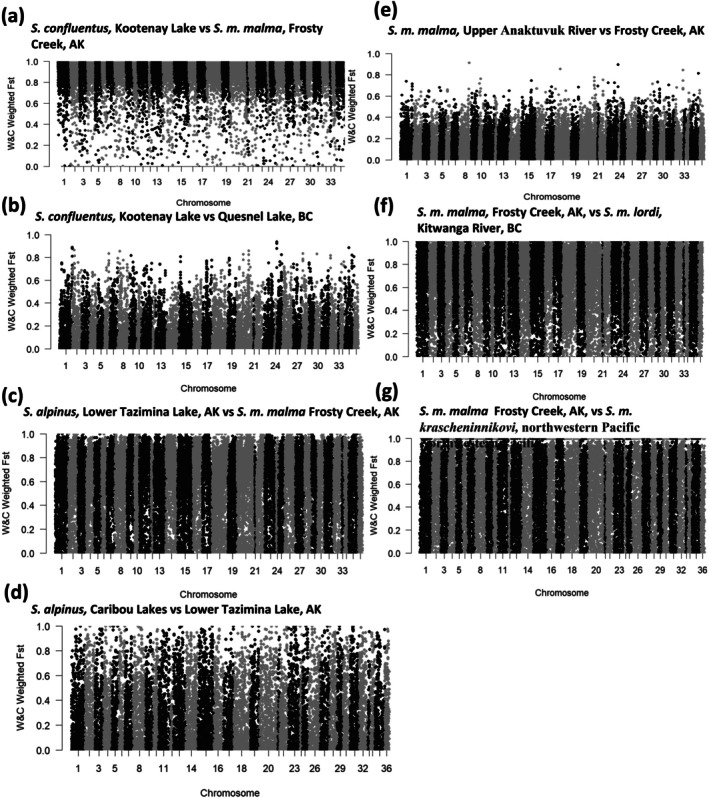
Manhattan plots of weighted mean *F*_ST_ (Weir and Cockerham 1984) across 36 autosomal chromosomes for different contrasts. (a) allopatric *S. confluentus* from Kootenay Lake, British Columbia (BC) and allopatric *S. m. malma* from Frosty Creek, Alaska AK (263,192 single nucleotide polymorphisms, SNPs), (b) allopatric *S. confluentus* from Kootenay and Quesnel lakes, BC (93,364 SNPs), (c) allopatric *S. m. malma* from Frosty Creek, AK, and allopatric *S. alpinus* from Lower Tazimina Lake, AK, (229,712 SNPs); (d) allopatric *S. alpinus* from Caribou Lakes and Lower Tazimina Lake, Alaska (70,475 SNPs); (e) *S. m. malma* from an unnamed tributary of the Upper Anaktuvuk River and Frosty Creek, Alaska (93,175 SNPs); (f) allopatric *S. m. malma* from Frosty Creek, AK, and *S. m. lordi* from the Kitwanga River, BC (187,412 SNPs; fish admixed with *S. confluentus* removed), and (g) *S. m. malma* from and unnamed tributary of the Upper Anaktuvuk River, AK, and *S. m. krascheninnikovi* from several localities in the northwestern Pacific (41,861 SNPs; see Table S1). Values of *F*_ST_ were calculated across 100 kb sliding windows with a window step size of 25 kb.

**FIGURE A2:**
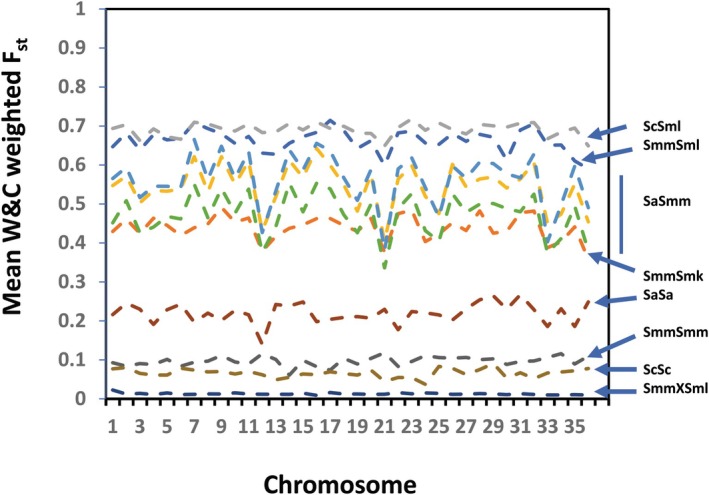
Weighted mean *F*_ST_ (Weir and Cockerham 1984) averaged within each of 36 autosomal chromosomes for different contrasts. ScSml, *Salvelinus confluentus* and *S. malma* *lordi* from the Kitwanga River, British Columbia; SmmSml, *S. m. malma* and *S. m. lordi* from several Alaskan lakes (see Table S1); SaSmm *S. alpinus* and *S. m. malma* from Lake Aleknagik, Iliamna Lake, and Lake Nerka, Alaska; SmmSmk, *S. m. malma* and *S. m. krascheninnikovi* from several localities in the northwestern Pacific (see Table S1); SaSa, *S. alpinus* from Caribou Lakes and Lower Tazimina Lake, Alaska; SmmSmm, *S. m. malma* from an unnamed tributary of the Upper Anaktuvuk River and Frosty Creek, Alaska; ScSc, *S. confluentus* from Kootenay and Quesnel lakes, British Columbia; SmmXSml, admixed *S. m. malma* and *S. m. lordi* from Chignik Lake, Alaska. Dashed lines are used to enhance clarity of presentation and do not imply any trend with chromosome number. Values of *F*_ST_ were calculated across 100 kb sliding windows with a window step size of 25 kb.

The Admixture analysis of the Kitwanga River sympatric *S. confluentus* and *S. m. lordi* clearly resolved the two species, but also the presence of several admixed samples (Figure 2; Table A1). Three of 13 admixed fish showed a bias towards *S. m lordi* (i.e., Q_*S. m. lordi*_ > 0.51, Figure 2); all other admixed fish were between Q_*S. m. lordi*_ = 0.49 and 0.51 suggesting that they were *F*_1_ hybrids. The triangle plot indicated that the sympatric *Salvelinus* of the Kitwanga River consisted mostly of unadmixed *S. confluentus* (*N* = 24) and *S. m. lordi* (*N* = 25; Figure 2; Table A2). Ten fish (77% of hybrids), however, were identified as likely *F*_1_ hybrids (i.e., those with a hybrid index of ~0.5 and an interclass heterozygosity of ~1.0; Table A2) and two fish (15% of hybrids) appeared to be backcrosses to *S. m. lordi* and only one (8%) was a later generation hybrid. Weir and Cockerham's (1984) average weighted *F*_ST_ was 0.71 between sympatric *S. confluentus* and *S. m. lordi*, with a distribution tending towards higher values consistently across the genome (Figures 2 and 3‐ScSmlK). By contrast, *F*_ST_ between an allopatric population of *S. confluentus* and an allopatric population of *S. m. malma* was 0.90 (Figure 3‐ScKLSmmFC, Figure A1). Two allopatric populations of *S. confluentus* had much lower average *F*_ST_ (0.07; Figure 3‐ScSc) as did two allopatric samples of *S. m. malma* (*F*_ST_ = 0.10, respectively, Figure 3‐SmmSmm, Figures A1 and A2; we did not have sufficient samples of allopatric *S. m. lordi* for comparison). Measures of *d*_xy_ were in general agreement in a relative sense with the exception that the decrease in *d*_xy_ between sympatric *S. confluentus* and *S. m. lordi* compared to the allopatric contrast was lower than when evaluated with *F*_ST_ (6.5% lower vs. 10%, Figure 3). Nucleotide diversity was lower in *S. confluentus* than *S. malma*, both in sympatry and allopatry (Table A3).

### 3.2 *Salvelinus alpinus* and *S. m. malma*

**FIGURE 4:**
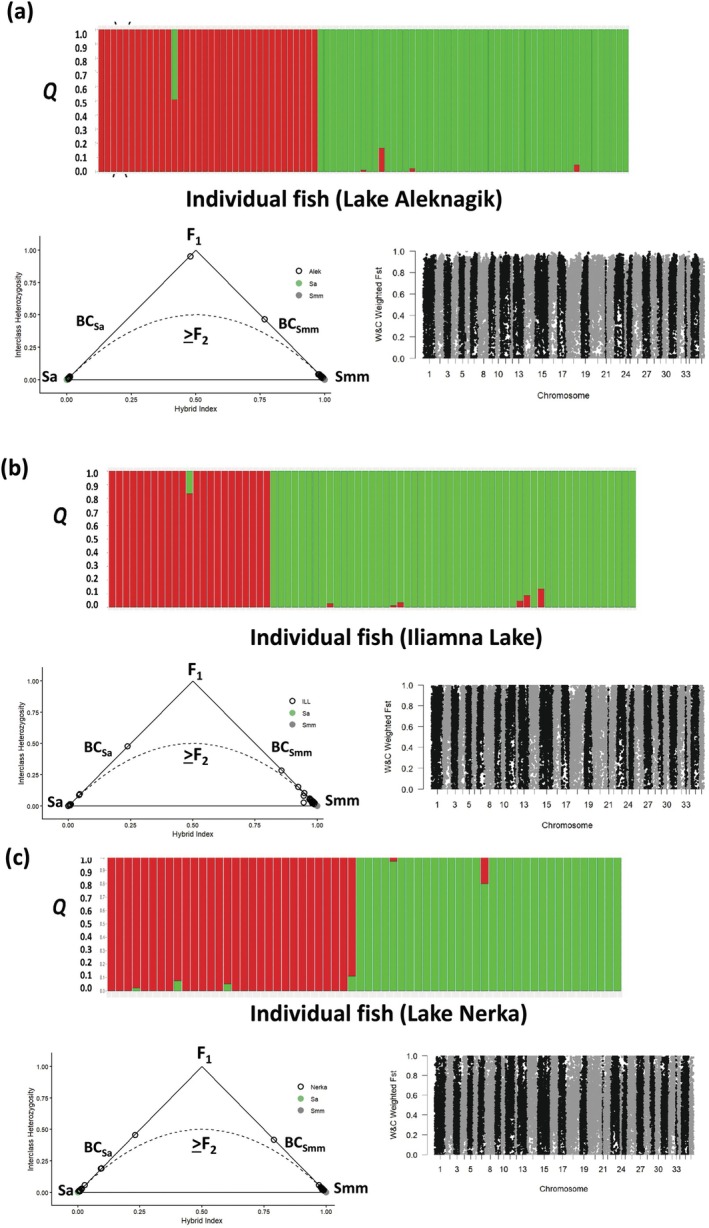
Differentiation between sympatric *Salvelinus alpinus* and *S. malma* *malma* from (a) Lake Aleknagik, (b) Iliamna Lake, and (c) Lake Nerka, southwestern Alaska, examined by (upper for each lake) unsupervised Admixture (Alexander et al. 2009), Triangle plot analysis (lower left for each lake), and weighted mean Weir and Cockerham's (1984) *F*_ST_ (lower right for each lake). In Admixture plots the *y*‐axis is the proportion of ancestry (*Q*) within *K* = 2 genetic groups (red = *S. alpinus* , green = *S. m. malma*) for each individual fish (thin vertical lines) inferred from between 22,163 and 26,183 single nucleotide polymorphisms. Metrics for models for *K* = 1–5 can be found in Table A1. The Triangle Plots show projection of interclass heterozygosity by hybrid index (0 = *S. alpinus* , 1.0 = *S. m. malma*), estimated using unlinked loci with fixed differences between parental taxa. Sa = parental samples of *S. alpinus* (green points in lower left), Smm = parental samples of *S. m. malma* (gray points in lower right); samples from each of the three lakes are open circles. Approximate positions along the triangle or the dotted line show those expected for *S. alpinus* (Sa), *S. m. malma* (Smm), *F*_1_ hybrids between *S. alpinus* and *S. m. malma*, *F*_2_ and later generation hybrids (≥ *F*_2_) and backcross generation 1 to *S. alpinus* (BC_Sa_) or *S. m. malma* (BC_Smm_). Successive backcross generations will occupy positions between BC_Sa_ and Sa (or BC_Smm_ and Smm). Many points overlap; individual scores are given in Table A4. The analysis of *F*_ST_ shows; see Manhattan plots of weighted mean *F*_ST_ (Weir and Cockerham 1984) across 36 autosomal chromosomes calculated across 100 kb sliding windows with a window step size of 25 kb using between 89,720 to 98,353 single nucleotide polymorphisms.

For the three lakes in southwestern Alaska, Admixture analyses resolved two distinct clusters of samples with little evidence of admixture between *S. alpinus* and *S. m. malma* (Figure 4; Table A1). In each of the three Alaskan lakes, the triangle plots identified most fish as either *S. alpinus* or *S. m. malma* and only one fish (Lake Aleknagik) was identified as a likely *F*_1_ hybrid between *S. alpinus* and *S. m. malma*. Rather, of the few hybrids found, most were suggested to be backcrosses or *F*_2_ or later generation hybrids (Figure 4; Table A4). Genome wide *F*_ST_ averaged between 0.56 and 0.61 in sympatric *S. alpinus* and *S. m. malma* from the three Alaskan lakes with a more even distribution between higher and lower values, again, spread consistently across the genome (Figure 3‐SaSmmA, SaSmmI, and SaSmmN, Figure 4). An allopatric population of *S. alpinus* and an allopatric population of *S. m. malma*, showed a very similar average *F*_ST_ (0.56, Figure 3‐SaLTSmmFC; Figure A1). By contrast, *F*_ST_ was lower between two allopatric populations of *S. alpinus* and also between two allopatric populations of *S. m. malma* (*F*_ST_ = 0.29 and 0.10, respectively, Figure 3‐SaSa, and SmmSmm; Figures A1 and A2). The estimates of *d*_xy_ were slightly lower in comparisons of sympatric *S. alpinus* and *S. m. malma* than in the comparison between allopatric populations of these two taxa; estimates were lower still in intrataxon comparisons (Figure 3). Nucleotide diversity was consistently lower in *S. alpinus* than *S*. *m. malma* (Table A3).

### 3.3 *S. m. malma* and *S. m. lordi*

**FIGURE 5:**
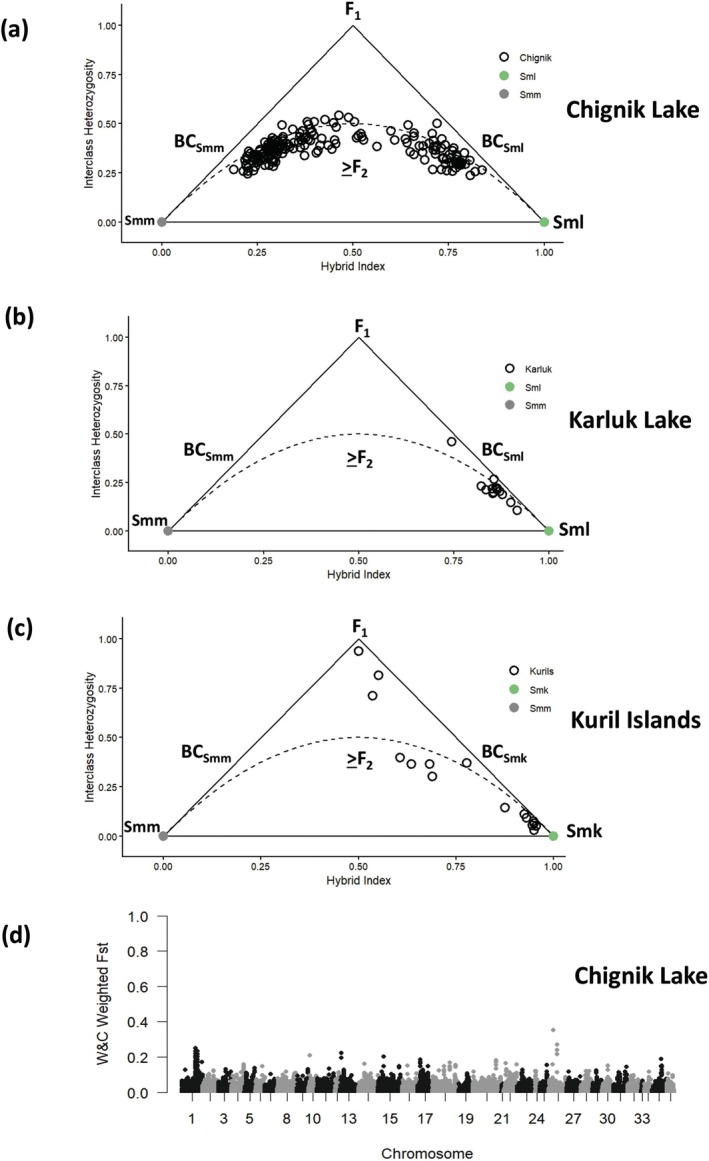
Triangle Plot analysis for admixed *Salvelinus malma* *malma* and *S. m. lordi* in (a) Chignik Lake, (b) Karluk Lake, both in southwestern Alaska, and (c) for *S. m. krascheninnikovi* and admixed *S. m. krascheninnikovi* and *S. m. malma* from the Kuril Islands, northwestern Pacific. Also shown is (d) weighted mean Weir and Cockerham's (1984) *F*_ST_ between admixed *S. m. malma* and *S. m. lordi* in Chignik Lake. The triangle plots in (a) and (b) show projection of interclass heterozygosity by hybrid index (0 = *S. m. malma*, 1.0 = *S. m. lordi*), estimated using unlinked loci with fixed differences between parental taxa. Smm = parental samples of *S. m. malma* (gray points in lower left), Sml = parental samples of *S. m. lordi* (green points in lower right); Chignik and Karluk lakes, and Kuril Islands samples are open circles. Approximate positions along the triangle or the dotted line show those expected for *S. m. malma* (Smm), *S. m. lordi* (Sml), *F*_1_ hybrids between *S. m. malma* and *S. m. lordi*, *F*_2_ and later generation hybrids (≥ *F*_2_) and backcross generation 1 to *S. m. malma* (BC_Smm_) or *S. m. lordi* (BC_Sml_). Successive backcross generations will occupy positions between BC_Smm_ and *S. m. malma* (or BC_Sml_ and *S. m. lordi*). Triangle plot in (c) shows the same points for *S. m. malma* and *S. m*. *krascheninnikovi* (Smk, green points in lower right) and all hybrid classes as above. Many points overlap; individual scores are given in Tables A5 and A6. The analysis of *F*_ST_ shows a Manhattan plot of weighted mean *F*_ST_ (Weir and Cockerham 1984) across 36 autosomal chromosomes calculated across 100 kb sliding windows with a window step size of 25 kb using 105,639 single nucleotide polymorphisms.

Triangle plots indicated that in Chignik and Karluk lakes, no parental genotypes of *S. m. malma* or *S. m. lordi* were observed; all fish were identified as post‐*F*_1_ hybrids or backcrosses to either parental taxon (Chignik Lake), or similar to *F*_2_ or later generation hybrids or backcrosses to *S. m. lordi* (Karluk Lake; Figure 5; Table A5). Average weighted *F*_ST_ was only 0.06 between admixed *S. m. malma‐*like (i.e., fish with Q_*S. m. malma*_ > 0.5) and *S. m. lordi‐*like (i.e., Q_*S. m. lordi*_ > 0.5) fish from the Chignik Lake watershed (Figure 3‐SmmXSml, Figure 5). By contrast, *F*_ST_ was substantially higher (= 0.67, Figure 3‐SmmSml) between two allopatric populations of *S. m. malma* and *S. m. lordi* with values concentrated at 0.70 and higher (Figures A1 and A2). Similar to comparative estimates of *F*_ST_, Measures of *d*_xy_ were lower in the admixed Chignik Lake watershed than in the comparison between allopatric *S. m. malma* and *S. m. lordi* (Figure 3). Nucleotide diversity in the admixed Chignik Lake watershed population was similar to that in allopatric populations of *S. m. malma* and *S. m. lordi* (Table A3).

### 3.4 *S. m*. *malma* and *S. m. krascheninnikovi*

In the samples from the Kuril Islands, the triangle plot identified fish as *S. m. krascheninnikovi* (but none as *S. m. malma*), *F*_1_ and post‐*F*_1_ hybrids and backcrosses to *S. m. krascheninnikovi* (Figure 5; Table A6). Between allopatric *S. m. krascheninnikovi* and *S. m. malma*, average weighted *F*_ST_ was 0.45 with high values spread across the genome (Figure 3‐SmmSmk, Figures A1 and A2). The estimated value of *d*_xy_ between allopatric *S. m. krascheninnikovi* and *S. m. malma* was lower than that between allopatric *S. m. malma* and *S. m. lordi* (Figure 3).

## 4 Discussion

How the extent and distribution of genetic differentiation across the genome proceeds along the speciation continuum, that is, a gradient of reproductive isolation, is an important question in speciation research as is the degree to which intrinsic genomic incompatibilities and/or extrinsic ecological factors, and how they vary in importance with time, contribute to speciation (e.g., Hendry et al. 2009; Stankowski and Ravinet 2021). Here, we examined the extent of hybridization and introgression, the kinds of hybrids produced, and genomic differentiation among a group of *Salvelinus* fishes that occur both in sympatry and allopatry. Further, we used their divergence times estimated from genomic data as a proxy for their position along a speciation continuum to better understand how aspects of reproductive isolation vary with time.

### 4.1 Divergence Time and Hybridization

Molecular estimates of divergence time have often been found to scale with measures of reproductive isolation. For instance, Coyne and Orr (1989) presented the foundational case of increasing reproductive isolation with increasing divergence time as inferred from genetic distance in *Drosophila*. Further, Singhal and Moritz (2013) found that reproductive isolation and divergence time were tightly coupled in ecologically cryptic forms of skinks, especially for those pairs estimated to have diverged more than 5 mya. By contrast, Moyle et al. (2004) found that the association between inferred divergence time and measures of reproductive isolation was weak or nonexistent in two of three genera of angiosperms. Still, reproductive isolation increasing with inferred divergence times is a common observation (e.g., Mallet et al. 2007; Mendelson 2003; Linan et al. 2021).

We found that of the two pairs of species examined, the pair that exhibited high numbers of *F*_1_ hybrids, as well two backcrosses and one older generation hybrid, was the pair with the oldest estimated divergence time– *S. confluentus* and *S. malma* (~5.2 million years ago, mya, Taylor et al. 2025; see also Lecaudey et al. 2018). Although our *S. confluentus* / *S. malma* contrast was limited to a single sympatric locality, this result appears consistent across time (Maier 2025) and is common to other localities (see Baxter et al. 1997; Redenbach and Taylor 2003)‐putative *F*_1_ and other classes of hybrids are regularly produced. By contrast, sympatric *S. alpinus* and *S. m. malma* from three southwestern Alaskan lakes showed much less evidence of contemporary hybridization as only a single *F*_1_ hybrid was identified, yet these species are estimated to have diverged only about 1.4 mya (McPhail 1961; Gharrett et al. 1991; May‐McNally et al. 2015; Lecaudey et al. 2018; Taylor et al. 2025). The low occurrence of post‐*F*_1_ hybrids and backcrosses in the sympatric *S. confluentus* and *S. m. lordi* contrast suggests that they show a high level of reproductive isolation likely through a combination of extrinsic and intrinsic postmating isolating processes that reduce the viability and/or fertility of *F*_1_ hybrids. By contrast, most hybrids between *S. alpinus* and *S. m malma* appeared to be backcrosses to either parental species. This suggests that while behavioral premating isolation and extrinsic ecological isolation appear to be well developed or at least likely (see below and Dennert et al. 2016), postmating intrinsic isolation may be less developed relative to that in *S. confluentus* and *S. m. lordi*. In this sense, the relationship between divergence time and reproductive isolation appears consistent with the ideas and studies reviewed by Coyne and Orr (1997). Here, data from examination of multiple pairs of *Drosophila* species indicated that although both premating and postmating isolation increased with time, the relationship between postmating isolation and reproductive isolation was twice as strong (Coyne and Orr 1997). In our system, *S. confluentus* and *S. m lordi* show more overlap in breeding locations, as a measure of premating isolation, in streams than the more recently derived *S. alpinus* and *S. m. malma* which breed in entirely different areas in sympatry; on submerged lake shore beaches, and in tributary streams, respectively (Weinstein et al. 2024 and see below). By contrast, our estimation of the number and kinds of hybrids produced suggest ongoing gene flow in *S. alpinus* and *S. m. malma*, but very much reduced effective gene flow in *S. confluentus* and *S. m. lordi*. Hence, postmating isolation in *Salvelinus* seems to track divergence time better than premating isolation.

The two subspecies *S. m. malma* and *S. m. lordi*, however, are thought to have diverged from a common ancestor only shortly after the split between *S. alpinus* and *S. malma* (~1.3 vs. 1.4 mya, Taylor et al. 2025). In at least two places where the subspecies come into contact, they appear to freely interbreed and form hybrid swarms (Taylor and May‐McNally 2015; Liu et al. 2024; current study). This suggests that premating and postmating isolation between two subspecies (*S. m. malma* and *S. m. lordi*) is very weak or absent, in stark contrast to what is observed in *S. alpinus* and *S. m. malma*, even though the divergence time between the subspecies is roughly equal to that between *S. m. malma* and *S. alpinus* . Finally, *Salvelinus* sampled from the Kuril Islands of the western North Pacific showed admixture between *S. m. malma* and *S. m. krascheninnikovi* with evidence of *F*_1_, *F*_2_ and later generation hybrids and backcrosses to *S. m. krascheninnikovi*. These subspecies appear to have diverged from each other more recently than any of the other taxon pairs examined (~0.8 mya, Taylor et al. 2025). Considered together, our data suggest that there may be a complex relationship between estimated divergence time and the extent of reproductive isolation and position along the speciation continuum at least in this group of fishes and over the time scale considered. It is also possible that divergence times among all taxa (Taylor et al. 2025) may be impacted by historical gene flow that complicates the relationship between divergence time and reproductive isolation as assessed here. Historical gene flow was estimated to have occurred among *S. confluentus* , *S. malma* (and its subspecies), and *S. alpinus* and was highest between the latter two taxa (Taylor et al. 2025). Consequently, historical gene flow could have compressed the divergence time estimate between *S. alpinus* and *S. malma* towards that between *S. m. malma and S. m. lordi* (Leaché et al. 2014).

### 4.2 Ecological Context and Hybridization

Differences in the ecological and geographic context of each paired comparison in *Salvelinus* may contribute to the relationship between inferred divergence times and the extent of reproductive isolation in these fishes. First, the degree of reproductive habitat segregation between sympatric *S. confluentus* and *S. m. lordi* appears to be less than between sympatric *S. alpinus* and *S. m. malma*. In the cases of the former two species, adults of both species use streams tributary to lakes or larger rivers for spawning although there may be some distinction in microhabitats used and time of spawning. Where sympatric, *S. confluentus* tend to be larger and spawn earlier (peak in Sept) than smaller *S. m. lordi* (peak in October; Bustard and Royea 1995; Leary and Allendorf 1997; Hagen and Taylor 2001; Mochnacz et al. 2013). In this case, overlapping reproductive habitat use may facilitate hybridization particularly if smaller, *S. m. lordi* males obtain “satellite” or “sneaker” spawning opportunities when found with larger *S. confluentus* male–female pairs (Baxter et al. 1997). A similar process appears to account for hybridization between *S. leucomaenis* (whitespotted char) and *S.* *malma* (the smaller species) in some northeastern Asian rivers (Yamamoto et al. 2006; Gruzdeva et al. 2020; Kuzishchin and Gruzdeva 2024). By contrast, sympatric *S. alpinus* and *S. m. malma* in our study area show greater reproductive habitat segregation than sympatric *S. confluentus* and *S. malma lordi*; *S. alpinus* spawn on submerged gravel beaches of lakes, whereas *S. m. malma* spawn in tributary streams (DeLacy and Morton 1943; Moriarty 1977; McBride 1980), differences that likely limit opportunities for hybridization.

Liu et al. (2024) and Taylor et al. (2025) showed that both in the Chignik and Karluk Lake watersheds, there were no *S. malma* characterized by Q_*S. m. malma*_ or Q_*S. m. lordi*_ of 1.0; all fish were admixed between *S. m. malma* and *S. m. lordi* (mean Q_*S. m. malma*_ = 0.51 in Chignik Lake and 0.149 in Karluk Lake). Consequently, contact between *S. m. malma* and *S. m. lordi* in these lakes results in hybrid swarms despite the fact that the subspecies are about as divergent from each other as are *S. alpinus* and *S. malma* (Taylor et al. 2025). Although sampl sizes from the Kuril Islands chain were small, we did find a few nonadmixed *S. m. krascheninnikovi* as well as several fish that showed appreciable admixture with *S. m. malma*.

There are no *S. alpinus* recorded from Chignik Lake and *S. malma* in that watershed use a wide range of lake, stream, estuarine and ocean habitats (Bond et al. 2014). Regardless, in Chignik and Karluk lakes, *S. m. malma‐*like and *S. m. lordi‐*like char appear to be stream‐spawners only. Indeed, although *S. m. malma* and *S. m. lordi* may occur in lakes during other phases of their life cycle, they appear to require running water for spawning, and spawning in habitats within lakes per se (e.g., submerged wave‐action exposed beaches) is not known to occur (McPhail 2007; Quinn 2018; Weinstein et al. 2024). In addition, in western Alaskan sympatric populations *S. alpinus* and *S. malma* appear to be ecologically specialized to distinct migratory and feeding niches—lake‐dwelling versus sea‐run, respectively (DeLacy and Morton 1943; Dennert et al. 2016). Even in sympatry and where both *S. alpinus* and *S. malma* are sea‐run, they display distinctions in migratory behavior and in the spatial distribution of overwintering sites (Smith et al. 2025a, 2025b). By contrast, there appears to be no such clear distinction between *S. m. malma* and *S. m. lordi*, particularly when they come into contact (DeLacy and Morton 1943; Bond et al. 2014; Liu et al. 2024; Weinstein et al. 2024). Consequently, the apparent similarity in the ecological attributes of *S. m. malma* and *S. m. lordi* may result in greater spatial overlap during breeding and little selection against hybrids between the two, thus promoting the formation of hybrid swarms.

The geographic context of the Kuril Islands and adjacent areas of the northwestern Pacific samples of *Salvelinus* is distinct in that we sampled no known areas of sympatry between *S. m. malma* and *S. m*. *krascheninnikovi*. Rather, all samples came from a series of streams from Sakhalin Island east and north through the Kuril Island chain. This area is considered just beyond the southern margin of the range of *S. m. malma* and, accordingly, we found no parental genotypes of that subspecies although our sample size was small. Most fish were either parental *S. m. krascheninnikovi* or *S. m. krascheninnikovi‐*like fish with some admixture with *S. m. malma* which is consistent with earlier work using allozymes (Omelchenko et al. 2002) and with the sampled area being in the heart of the range of *S. m. krascheninnikovi*. The range boundary between the two subspecies is thought to lie somewhere between the northern Kuril Islands and the southern tip of the Kamchatka Peninsula (near Shumshu Island, Gritsenko et al. 1998; Omelchenko et al. 2002). Our finding of hybrids in more southern islands (including likely *F*_1_ hybrids on Urup, Onekotan, and Kunashir islands) using Triangle Plot analysis, however, suggests that *S. m. malma* may occur, perhaps in small numbers, in the Kuril Islands. Alternatively, hybrids in salmonid fishes tend to stray more than parental species or populations which may account for the presence of *F*_1_ hybrids well south of the presumed contact zone (Candy and Beacham 2000; Bourret et al. 2022). Similarly, admixture between *S. m. malma* and *S. m. lordi* in Chignik Lake watershed was balanced between the subspecies (Liu et al. 2024) while that in the Karluk Lake watershed was strongly biased towards *S. m. lordi* (Taylor et al. 2025). This difference between the watersheds is likely driven by the position of Chignik Lake on the Alaska Peninsula in the heart of the contact zone between *S. m. malma* and *S. m. lordi*, whereas Karluk Lake is closer to the heart of the distribution of *S. m. lordi* (Taylor and May‐McNally 2015; Weinstein et al. 2024). Further, and as with the case of *S. m. malma and S. m. lordi* in southwestern Alaska, general similarities in the ecology of *S. m. malma* and *S. m. krascheninnikovi* (Armstrong and Morrow 1980; Weinstein et al. 2024) likely promote hybridization between them when in contact (cf. Omelchenko et al. 2002; Radchenko 2004; Yamamoto et al. 2021). Overall, our analyses point to the importance of ecological distinctions between coexisting taxa, and to a lesser extent the degree of geographic overlap, as being critical factors that appear to influence the degree and patterns of introgressive hybridization within North Pacific *Salvelinus* despite a 3 to 6‐fold difference in inferred divergence times (5.2 mya between *S. confluentus* and *S. malma* vs. 1.4 mya between *S. alpinus* and *S. malma* vs. 0.8 mya between *S. m. malma* and *S. m*. *krascheninnikovi*; Taylor et al. 2025).

### 4.3 Genomic Landscape of Divergence

Our genome wide assessments of the degree of genetic differentiation as represented by *F*_ST_ indicated two broad patterns. First, both in contrasts between sympatric *S. confluentus* and *S. m. lordi* and between *S. alpinus and S. m. malma*, divergence was consistently high across the genome. Average *F*_ST_ and *d*_xy_ were higher in the *S. confluentus* and *S. m. lordi* contrast relative to that between *S. alpinus and S. m. malma* which is consistent with the 3‐fold longer divergence time between the former pair. Hence, both pairs of species comparisons would seem to indicate that they are well advanced along the speciation continuum (*sensu* Stankowski and Ravinet 2021, figure 2; see also Seehausen et al. 2014) such that restrictions to gene flow, while incomplete, are widespread across the genome (see also Wu and Ting 2004). Second, and what is remarkable in *Salvelinu*s is that such advanced reproductive isolation in one pair of taxa ( *S. alpinus* and *S. m. malma*) contrasts starkly with an apparent lack of reproductive isolation between another (*S. m. malma* and *S. m lordi*) even though the inferred divergence times of both pairs are similar (1.4 vs. 1.3 mya with overlapping credible limits, Taylor et al. 2025). Again, the contrast in ecological differences (extensive between *S. alpinus* and *S. m. malma* vs. essentially absent between *S. m. malma* and *S. m. lordi*) suggests the strong role that ecology may play in influencing the outcomes of contact between taxa, via pre‐ and/or postmating isolation, and perhaps in contributing to rapid speciation (i.e., the difference in estimated divergence times between these pairs of taxa was 100,000 years or less). Indeed, the observation that differences between coexisting *Salvelinus* taxa (or lack thereof) in ecological aspects of breeding habitat is consistent with the idea that divergent selection driving premating isolation may initiate reproductive isolation along the speciation continuum in some contexts (Coyne and Orr 1989; Seehausen et al. 2014). Differences in migratory behavior, size, and feeding ecology between *S. confluentus* and *S. malma* and between *S. alpinus* and *S. malma* (DeLacy and Morton 1943; Hagen and Taylor 2001; May‐McNally et al. 2015; Dennert et al. 2016; Smith et al. 2025a, 2025b), however, leave open the possibility that extrinsic postmating processes are also important in reproductive isolation in these fishes. The role of intrinsic postmating factors in initiating or enhancing reproductive isolation remains unexplored in these taxa although such factors have been shown to be present between recently‐derived sympatric and partially reproductively isolated ecotypes of brook trout, *S. fontinalis* (cf. Mavarez et al. 2009).

Taylor et al. (2025) presented evidence for a model of divergence in allopatry in *S. alpinus* and *S. malma* accompanied by some gene flow following secondary contact. This raises the hypothesis that divergence in isolation “primed” reproductive isolation and the presence of ecological opportunity (reproductive habitat in streams versus lakes) in sympatry has maintained strong reproductive isolation. Indeed, sympatry may have enhanced reproductive isolation via reinforcement, but isolation remains incomplete even in the older divergence between *S. confluentus* and *S. malma* (this study; cf. Stankowski et al. 2020).

In conclusion, we used a comparative genomic approach to better understand the relationship between inferred divergence times and the degree of reproductive isolation between taxa within North Pacific *Salvelinus* fishes. Our results showed that even after up to just over 5 million years of divergence, reproductive isolation, while advanced in some pairs of taxa, is incomplete. Further, our analyses support the idea that ecological attributes, particularly those relevant to premating isolation, are key factors that influence outcomes of contact between taxa and, hence, contribute to the relationship between divergence time and position along the speciation continuum (Hernández‐Hernández et al. 2021; Stankowski and Ravinet 2021).

## Author Contributions

**E. B. Taylor:** conceptualization (equal), data curation (lead), formal analysis (equal), funding acquisition (lead), investigation (equal), methodology (equal), project administration (lead), resources (lead), visualization (equal), writing – original draft (lead). **A. Geraldes:** conceptualization (equal), formal analysis (equal), investigation (equal), methodology (equal), resources (equal), writing – review and editing (equal).

## Funding

The work was funded by Natural Sciences and Engineering Research Council of Canada Discovery (8‐9616) and Equipment (3588868‐2008) grants.

## Conflicts of Interest

The authors declare no conflicts of interest.

## Supporting information


**Table S1:** List of all samples used in analyses.

## Data Availability

Raw (unfiltered and demultiplexed) variant call format files used as the basis for all analyses are available on the National Center for Biotechnology Information's (NCBI's) Short Read Archive (SRA) under BioProjects PRJNA1116813, PRJNA1299464, and PRJNA1389838.

## Appendix 1

**TABLE A1:** Cross validation errors (CVE) produced by Admixture's (Alexander et al. 2009) clustering algorithm applied to sympatric populations of *Salvelinus confluentus* and *S. malma lordi* from one locality, and *Salvelinus alpinus* and *S.* *malma malma* from three localities.

*K*	*S. confluentus* and *S. m. lordi* Kitwanga River, BC	*S. alpinus* and *S. m. malma*: Aleknagik Lake, AK	*S. alpinus* and *S. m. malma*: Iliamna Lake, AK	*S. alpinus* and *S. m. malma*: Lake Nerka, AK
1	0.52038	0.34305	0.37932	0.41697
2	**0.25461**	**0.23994**	**0.29526**	**0.26758**
3	0.28758	0.25325	0.31194	0.29823
4	0.31276	0.27103	0.34434	0.32932
5	0.33442	0.29504	0.37167	0.35491

*Note:* All values are the means across 5 runs and *K* = the putative number of genetic groups. Boldface values indicate the favored *K* according to the lowest CVE.

Abbreviations: AK, Alaska, US; BC, British Columbia, Canada.

**TABLE A2:** Results of triangulaR analyses (Wiens et al. 2025) for allopatric *Salvelinus confluentus* (Sc) and allopatric *S. malma lordi* (Sml) and sympatric populations from the Kitwanga River (KIT), British Columbia. Sample ID codes are defined in Table S1.

Sample ID	Taxon	Hybrid index	Interclass heterozygosity	Proportion missing data
BT_AK_NN_A04_571	Sc	0.0000	0.0000	0.0503
BT_AK_NN_A04_572	Sc	0.0000	0.0000	0.2585
BT_AK_NN_A04_573	Sc	0.0000	0.0000	0.0626
BT_AK_NN_A04_574	Sc	0.0000	0.0000	0.0970
BT_AK_NN_A04_575	Sc	0.0000	0.0000	0.0724
BT_AK_NN_A04_576	Sc	0.0000	0.0000	0.0775
BT_AK_NN_A04_577	Sc	0.0000	0.0000	0.2519
BT_AK_NN_A04_579	Sc	0.0000	0.0000	0.0868
BT_AK_NN_A04_580	Sc	0.0000	0.0000	0.0896
BT_AK_NN_A04_581	Sc	0.0000	0.0000	0.1042
BT_AK_NN_A04_582	Sc	0.0000	0.0000	0.0584
BT_AK_NN_A04_583	Sc	0.0000	0.0000	0.0503
BT_AK_NN_A04_584	Sc	0.0000	0.0000	0.0567
BT_AK_NN_A04_585	Sc	0.0000	0.0000	0.0640
BT_AK_NN_A04_586	Sc	0.0000	0.0000	0.0645
BT_AK_NN_A04_587	Sc	0.0000	0.0000	0.0605
EPSDV_BC_AS_A01_542	Sml	1.0000	0.0000	0.0854
EPSDV_BC_AS_A01_543	Sml	1.0000	0.0000	0.2812
EPSDV_BC_AS_A01_544	Sml	1.0000	0.0000	0.0831
EPSDV_BC_AS_A01_545	Sml	1.0000	0.0000	0.0816
EPSDV_BC_AS_A01_546	Sml	1.0000	0.0000	0.0773
EPSDV_BC_AS_A01_547	Sml	1.0000	0.0000	0.1072
EPSDV_BC_AS_A01_548	Sml	1.0000	0.0000	0.0564
EPSDV_BC_AS_A01_549	Sml	1.0000	0.0000	0.0569
EPSDV_BC_AS_A02_550	Sml	1.0000	0.0000	0.2306
EPSDV_BC_AS_A02_551	Sml	1.0000	0.0000	0.2004
EPSDV_BC_AS_A02_552	Sml	1.0000	0.0000	0.1333
EPSDV_BC_AS_A02_553	Sml	1.0000	0.0000	0.4565
EPSDV_BC_AS_A02_554	Sml	1.0000	0.0000	0.2385
EPSDV_BC_AS_A02_555	Sml	1.0000	0.0000	0.1654
EPSDV_BC_AS_A02_556	Sml	1.0000	0.0000	0.6023
xBTEPSDV_BC_NN_A04_897	KIT	0.4986	0.9702	0.0835
xBTEPSDV_BC_NN_A04_898	KIT	0.6088	0.0234	0.5991
xBTEPSDV_BC_NN_A04_899	KIT	0.4975	0.9643	0.0884
xBTEPSDV_BC_NN_A04_900	KIT	0.4994	0.9665	0.0623
xBTEPSDV_BC_NN_A04_901	KIT	0.7553	0.4696	0.1079
xBTEPSDV_BC_NN_A04_902	KIT	0.6956	0.5878	0.2217
xBTEPSDV_BC_NN_A04_903	KIT	0.4980	0.9688	0.0517
xBTEPSDV_BC_NN_A04_904	KIT	0.4973	0.9658	0.0535
xBTEPSDV_BC_NN_A04_905	KIT	0.4985	0.9530	0.0839
xBTEPSDV_BC_NN_A04_906	KIT	0.4987	0.9638	0.0639
xBTEPSDV_BC_NN_A04_907	KIT	0.4970	0.9657	0.1411
xBTEPSDV_BC_NN_A04_908	KIT	0.5008	0.9705	0.0607
xBTEPSDV_BC_NN_A04_909	KIT	0.4982	0.9629	0.0736
BT_BC_NN_A04_588	KIT	0.0035	0.0037	0.0714
BT_BC_NN_A04_589	KIT	0.0045	0.0056	0.0500
BT_BC_NN_A04_590	KIT	0.0047	0.0046	0.0790
BT_BC_NN_A04_591	KIT	0.0033	0.0031	0.0698
BT_BC_NN_A04_592	KIT	0.0036	0.0032	0.0440
BT_BC_NN_A04_593	KIT	0.0036	0.0032	0.0587
BT_BC_NN_A04_594	KIT	0.0078	0.0113	0.0956
BT_BC_NN_A04_595	KIT	0.0044	0.0051	0.0763
BT_BC_NN_A04_596	KIT	0.0109	0.0178	0.1026
BT_BC_NN_A04_597	KIT	0.0035	0.0035	0.0467
BT_BC_NN_A04_598	KIT	0.0054	0.0072	0.0653
BT_BC_NN_A04_599	KIT	0.0046	0.0050	0.0654
BT_BC_NN_A04_600	KIT	0.0034	0.0029	0.1625
BT_BC_NN_A04_601	KIT	0.0040	0.0046	0.0598
BT_BC_NN_A04_602	KIT	0.0035	0.0031	0.0705
BT_BC_NN_A04_603	KIT	0.0050	0.0052	0.1050
BT_BC_NN_A04_604	KIT	0.0098	0.0140	0.0835
BT_BC_NN_A04_605	KIT	0.0036	0.0036	0.0649
BT_BC_NN_A04_606	KIT	0.0055	0.0073	0.0943
BT_BC_NN_A04_607	KIT	0.0036	0.0034	0.0643
BT_BC_NN_A04_608	KIT	0.0088	0.0132	0.2818
BT_BC_NN_A04_609	KIT	0.0036	0.0028	0.0447
BT_BC_NN_A04_610	KIT	0.0036	0.0038	0.0837
BT_BC_NN_A04_611	KIT	0.0053	0.0071	0.0755
EPSDV_BC_AN_A04_517	KIT	0.9979	0.0030	0.1370
EPSDV_BC_AN_A04_518	KIT	0.9965	0.0067	0.1479
EPSDV_BC_AN_A04_519	KIT	0.9949	0.0094	0.1140
EPSDV_BC_AN_A04_520	KIT	0.9971	0.0052	0.0506
EPSDV_BC_AN_A04_521	KIT	0.9979	0.0034	0.0569
EPSDV_BC_AN_A04_522	KIT	0.9975	0.0045	0.1446
EPSDV_BC_AN_A04_523	KIT	0.9955	0.0082	0.0640
EPSDV_BC_AN_A04_524	KIT	0.9963	0.0069	0.1860
EPSDV_BC_AN_A04_525	KIT	0.9980	0.0034	0.0413
EPSDV_BC_AN_A04_526	KIT	0.9977	0.0037	0.0820
EPSDV_BC_AN_A04_527	KIT	0.9975	0.0040	0.0860
EPSDV_BC_AN_A04_528	KIT	0.9977	0.0038	0.2107
EPSDV_BC_AN_A04_529	KIT	0.9963	0.0067	0.2389
EPSDV_BC_AN_A04_530	KIT	0.9970	0.0045	0.0561
EPSDV_BC_AN_A04_531	KIT	0.9984	0.0029	0.1406
EPSDV_BC_AN_A04_532	KIT	0.9982	0.0030	0.0335
EPSDV_BC_AN_A04_533	KIT	0.9966	0.0050	0.2096
EPSDV_BC_AN_A04_534	KIT	0.9978	0.0041	0.2301
EPSDV_BC_AN_A04_535	KIT	0.9976	0.0038	0.0277
EPSDV_BC_AN_A04_536	KIT	0.9974	0.0050	0.1478
EPSDV_BC_AN_A04_537	KIT	0.9973	0.0048	0.0563
EPSDV_BC_AN_A04_538	KIT	0.9965	0.0061	0.1345
EPSDV_BC_AN_A04_539	KIT	0.9981	0.0028	0.0569
EPSDV_BC_AN_A04_540	KIT	0.9979	0.0036	0.0593
EPSDV_BC_AN_A04_541	KIT	0.9982	0.0033	0.0826

**TABLE A3:** List of nucleotide diversity (*π*) statistics in samples of *Salvelinus* used in comparative *F*_ST_ and *d*_xy_ analyses. Values are means calculated across 100 kb sliding windows with a window step size of 25 kb.

Sample	Mean *π*
Kitwanga River *S. confluentus*	0.000252
Kitwanga River *S. malma lordi*	0.001163
Allopatric Kootenay Lake *S. confluentus*	0.000498
Allopatric Quesnel Lake *S. confluentus*	0.000498
Allopatric Frosty Creek *S. m. malma*	0.001124
Allopatric Upper Anaktuvuk River *S. m. malma*	0.000637
Aleknagik Lake *S. alpinus*	0.000435
Aleknagik Lake *S. m. malma*	0.000905
Iliamna Lake *S. alpinus*	0.000287
Iliamna Lake *S. m. malma*	0.000617
Lake Nerka *S. alpinus*	0.000303
Lake Nerka *S. m. malma*	0.000726
Allopatric Caribou Lakes *S. alpinus*	0.000406
Allopatric Lower Tazimina Lake *S. alpinus*	0.000574
Chignik Lake admixed *S. m. malma* x *S. m. lordi*	0.001048
Northwestern Pacific *S. m*. *krascheninnikovi*	0.000734

**TABLE A4:** Results of triangulaR analyses (Wiens et al. 2025) for allopatric *Salvelinus* *malma malma* (Smm), allopatric *S. alpinus* (Sa), and sympatric populations from Lake Aleknagik (Alek, A), Iliamna Lake (ILL, B), and Lake Nerka (Nerka, C), Alaska. Sample ID codes are defined in Table S1.

(A) Sample ID	Taxon	Hybrid index	Interclass heterozygosity	Proportion missing data
AC_AK_AF_A01_011a	Sa	0.00000	0.00000	0.15521
AC_AK_AF_A01_012	Sa	0.00000	0.00000	0.09921
AC_AK_AF_A01_013	Sa	0.00000	0.00000	0.10806
AC_AK_AF_A01_014b	Sa	0.00000	0.00000	0.07957
AC_AK_AF_A01_015c	Sa	0.00000	0.00000	0.40472
AC_AK_AF_A01_016d	Sa	0.00000	0.00000	0.09037
AC_AK_AF_A01_017	Sa	0.00000	0.00000	0.21218
AC_AK_AF_A02_018	Sa	0.00000	0.00000	0.19450
AC_AK_AF_A02_019	Sa	0.00000	0.00000	0.18762
AC_AK_AF_A02_020	Sa	0.00000	0.00000	0.36542
AC_AK_AF_A02_021	Sa	0.00000	0.00000	0.38802
AC_AK_AF_A04_022	Sa	0.00000	0.00000	0.24361
AC_AK_AF_A04_023	Sa	0.00000	0.00000	0.09921
AC_AK_AF_A04_024	Sa	0.00000	0.00000	0.04224
AC_AK_AF_A04_025	Sa	0.00000	0.00000	0.07957
AC_AK_AF_A04_026	Sa	0.00000	0.00000	0.05992
AC_AK_AF_A04_027	Sa	0.00000	0.00000	0.06778
AC_AK_AF_A04_028	Sa	0.00000	0.00000	0.09037
AC_AK_AF_A04_029	Sa	0.00000	0.00000	0.06189
AC_AK_AF_A04_030	Sa	0.00000	0.00000	0.04813
AC_AK_AF_A04_031	Sa	0.00000	0.00000	0.07859
AC_AK_AF_A04_032	Sa	0.00000	0.00000	0.08644
AC_AK_AF_A04_033	Sa	0.00000	0.00000	0.07564
AC_AK_AF_A04_034	Sa	0.00000	0.00000	0.04813
AC_AK_AF_A04_035	Sa	0.00000	0.00000	0.32220
AC_AK_AF_A04_036	Sa	0.00000	0.00000	0.06189
AC_AK_AF_A04_037	Sa	0.00000	0.00000	0.08448
AC_AK_AF_A04_038	Sa	0.00000	0.00000	0.08546
AC_AK_AF_A04_039e	Sa	0.00000	0.00000	0.08153
AC_AK_AF_A04_040e	Sa	0.00000	0.00000	0.09528
AC_AK_AF_A04_041	Sa	0.00000	0.00000	0.05403
AC_AK_AF_A04_042	Sa	0.00000	0.00000	0.06287
AC_AK_AF_A04_043	Sa	0.00000	0.00000	0.06876
AC_AK_AF_A04_044	Sa	0.00000	0.00000	0.07760
AC_AK_AF_A04_045	Sa	0.00000	0.00000	0.10314
AC_AK_AF_A04_046	Sa	0.00000	0.00000	0.08546
AC_AK_AF_A04_047	Sa	0.00000	0.00000	0.05599
AC_AK_AF_A04_048	Sa	0.00000	0.00000	0.06582
AC_AK_AF_A04_049	Sa	0.00000	0.00000	0.06582
AC_AK_AF_A04_050	Sa	0.00000	0.00000	0.15128
AC_AK_AF_A04_051b	Sa	0.00000	0.00000	0.05403
AC_AK_AF_A04_052	Sa	0.00000	0.00000	0.08743
AC_AK_AF_A04_053	Sa	0.00000	0.00000	0.05796
AC_AK_AF_A04_054d	Sa	0.00000	0.00000	0.09037
AC_AK_AF_A04_055c	Sa	0.00000	0.00000	0.05796
AC_AK_AF_A04_056	Sa	0.00000	0.00000	0.06778
AC_AK_AF_A04_057	Sa	0.00000	0.00000	0.10216
AC_AK_AF_A04_058	Sa	0.00000	0.00000	0.05501
AC_AK_AF_A04_059	Sa	0.00000	0.00000	0.16208
AC_AK_AF_A04_060	Sa	0.00000	0.00000	0.13360
AC_AK_AF_A04_061	Sa	0.00000	0.00000	0.05108
AC_AK_AF_A04_062	Sa	0.00000	0.00000	0.23870
AC_AK_AF_A04_064	Sa	0.00000	0.00000	0.04519
AC_AK_AF_A04_065	Sa	0.00000	0.00000	0.13654
AC_AK_AF_A04_066	Sa	0.00000	0.00000	0.23674
AC_AK_AF_A04_068	Sa	0.00000	0.00000	0.09136
AC_AK_AF_A04_069	Sa	0.00000	0.00000	0.08743
AC_AK_AF_A04_070	Sa	0.00000	0.00000	0.07073
AC_AK_AF_A04_071	Sa	0.00000	0.00000	0.08153
AC_AK_AF_A04_072	Sa	0.00000	0.00000	0.10020
AC_AK_AF_A04_073	Sa	0.00000	0.00000	0.19843
AC_AK_AF_A04_074	Sa	0.00000	0.00000	0.17191
AC_AK_AF_A04_075	Sa	0.00000	0.00000	0.05206
AC_AK_AF_A04_076	Sa	0.00000	0.00000	0.04322
AC_AK_AF_A04_077	Sa	0.00000	0.00000	0.05108
AC_AK_AF_A04_078	Sa	0.00000	0.00000	0.06483
AC_AK_AF_A04_079	Sa	0.00000	0.00000	0.08841
AC_AK_AF_A04_081	Sa	0.00000	0.00000	0.38605
AC_AK_AF_CHI_088a	Sa	0.00000	0.00000	0.12181
AC_AK_AF_LBF_089	Sa	0.00000	0.00000	0.35069
AC_AK_AF_LBF_090	Sa	0.00000	0.00000	0.24067
AC_AK_AF_LBF_091	Sa	0.00000	0.00000	0.49214
AC_AK_AF_LBF_092	Sa	0.00000	0.00000	0.37328
AC_AK_AF_LBF_093	Sa	0.00000	0.00000	0.35363
AC_AK_AF_LBF_094	Sa	0.00000	0.00000	0.13065
AC_AK_AF_LBF_095	Sa	0.00000	0.00000	0.54322
AC_AK_AF_LBF_096	Sa	0.00000	0.00000	0.57859
NDV_AK_AF_A01_250	Smm	1.00000	0.00000	0.68369
NDV_AK_AF_A01_251	Smm	1.00000	0.00000	0.28684
NDV_AK_AF_A01_252	Smm	1.00000	0.00000	0.41257
NDV_AK_AF_A02_253	Smm	1.00000	0.00000	0.21120
NDV_AK_AF_A02_254	Smm	1.00000	0.00000	0.23183
NDV_AK_AF_A02_255	Smm	1.00000	0.00000	0.22692
NDV_AK_AF_A04_256	Smm	1.00000	0.00000	0.18861
NDV_AK_AF_A04_258	Smm	1.00000	0.00000	0.04322
NDV_AK_AF_A04_259	Smm	1.00000	0.00000	0.05403
NDV_AK_AF_A04_260	Smm	1.00000	0.00000	0.04519
NDV_AK_AF_A04_261	Smm	1.00000	0.00000	0.06385
NDV_AK_AF_A04_262	Smm	1.00000	0.00000	0.09627
NDV_AK_AF_A04_263	Smm	1.00000	0.00000	0.07466
NDV_AK_AF_A04_264	Smm	1.00000	0.00000	0.05108
NDV_AK_AF_A04_265	Smm	1.00000	0.00000	0.05796
NDV_AK_AF_A04_266	Smm	1.00000	0.00000	0.09921
NDV_AK_AF_A04_267	Smm	1.00000	0.00000	0.09725
NDV_AK_AF_A04_268	Smm	1.00000	0.00000	0.04420
NDV_AK_AF_A04_269	Smm	1.00000	0.00000	0.02947
NDV_AK_AS_A01_270	Smm	1.00000	0.00000	0.04617
NDV_AK_AS_A01_271h	Smm	1.00000	0.00000	0.35265
NDV_AK_AS_A02_272	Smm	1.00000	0.00000	0.21513
NDV_AK_AS_A02_273	Smm	1.00000	0.00000	0.26621
NDV_AK_AS_A04_274	Smm	1.00000	0.00000	0.10904
NDV_AK_AS_A04_275	Smm	1.00000	0.00000	0.19548
NDV_AK_AS_A04_276	Smm	1.00000	0.00000	0.23870
NDV_AK_AS_A04_277	Smm	1.00000	0.00000	0.20334
NDV_AK_AS_A04_279	Smm	1.00000	0.00000	0.23379
NDV_AK_AS_A04_280	Smm	1.00000	0.00000	0.15619
NDV_AK_AS_A04_281	Smm	1.00000	0.00000	0.29273
NDV_AK_AS_A04_282	Smm	1.00000	0.00000	0.01670
NDV_AK_AS_A04_283	Smm	1.00000	0.00000	0.05599
NDV_AK_AS_A04_284	Smm	1.00000	0.00000	0.04322
NDV_AK_AS_A04_287	Smm	1.00000	0.00000	0.09921
NDV_AK_AS_A04_288	Smm	1.00000	0.00000	0.03536
NDV_AK_AS_A04_289	Smm	1.00000	0.00000	0.07957
NDV_AK_AS_A04_290	Smm	1.00000	0.00000	0.03340
NDV_AK_AS_A04_291	Smm	1.00000	0.00000	0.11395
NDV_AK_AS_A04_292	Smm	1.00000	0.00000	0.08841
NDV_AK_AS_A04_293	Smm	1.00000	0.00000	0.05305
NDV_AK_AS_A04_294	Smm	1.00000	0.00000	0.07957
NDV_AK_AS_A04_295	Smm	1.00000	0.00000	0.03733
NDV_AK_AS_A04_296	Smm	1.00000	0.00000	0.02456
NDV_AK_AS_A04_297	Smm	1.00000	0.00000	0.04715
NDV_AK_AS_A04_299	Smm	1.00000	0.00000	0.04028
NDV_AK_AS_A04_302	Smm	1.00000	0.00000	0.04322
NDV_AK_AS_A04_304	Smm	1.00000	0.00000	0.07171
NDV_AK_AS_A04_305	Smm	1.00000	0.00000	0.07269
NDV_AK_AS_A04_307	Smm	1.00000	0.00000	0.04912
NDV_AK_SS_A01_330	Alek	0.98478	0.02826	0.09627
NDV_AK_SS_A01_331	Alek	0.98861	0.02278	0.09430
NDV_AK_SS_A04_353	Alek	0.98937	0.01923	0.02947
NDV_AK_SS_A04_354	Alek	0.99139	0.01722	0.03045
NDV_AK_SS_A04_355	Alek	0.97983	0.04034	0.07466
NDV_AK_SS_A04_356	Alek	0.98914	0.01954	0.09528
NDV_AK_SS_A04_357	Alek	0.98550	0.02436	0.15324
NDV_AK_SS_A04_358	Alek	0.97807	0.03937	0.12672
NDV_AK_SS_A04_359	Alek	0.99331	0.00927	0.04617
NDV_AK_SS_A04_360	Alek	0.98778	0.02444	0.07564
NDV_AK_SS_A04_361	Alek	0.76671	0.46658	0.22102
NDV_AK_SS_A04_362	Alek	0.98816	0.01957	0.04617
NDV_AK_SS_A04_363	Alek	0.98772	0.02252	0.04028
NDV_AK_SS_A04_364	Alek	0.98686	0.02208	0.06582
NDV_AK_SS_A04_365	Alek	0.99425	0.01149	0.05992
NDV_AK_SS_A04_366	Alek	0.98385	0.03229	0.05697
NDV_AK_SS_A04_367	Alek	0.98760	0.01446	0.04912
NDV_AK_SS_A04_368	Alek	0.99178	0.01439	0.04420
NDV_AK_SS_A04_369	Alek	0.01104	0.02208	0.11002
NDV_AK_SS_LBF_451	Alek	0.00841	0.01423	0.24067
NDV_AK_SS_LBF_452	Alek	0.98830	0.02094	0.20236
NDV_AK_SS_LBF_453	Alek	0.98939	0.02122	0.25933
NDV_AK_SS_LBF_454	Alek	0.98578	0.02246	0.34381
NDV_AK_SS_LBF_455	Alek	0.97510	0.03873	0.28978
NDV_AK_SS_LBF_466	Alek	0.98540	0.02920	0.59627
AC_AK_SF_A01_097	Alek	0.01238	0.01830	0.08743
AC_AK_SF_A01_098	Alek	0.00804	0.01378	0.14440
AC_AK_SF_A02_107	Alek	0.01115	0.02231	0.25147
AC_AK_SF_A02_108	Alek	0.00802	0.01605	0.20432
AC_AK_SF_A04_117	Alek	0.00407	0.00203	0.03438
AC_AK_SF_A04_118	Alek	0.00524	0.01047	0.06189
AC_AK_SF_A04_119	Alek	0.47944	0.95022	0.09234
AC_AK_SF_A04_120	Alek	0.01184	0.02368	0.12868
AC_AK_SF_A04_121	Alek	0.00624	0.00832	0.05501
AC_AK_SF_A04_122	Alek	0.00624	0.00624	0.05501
AC_AK_SF_A04_123	Alek	0.00877	0.01342	0.04813
AC_AK_SF_A04_124	Alek	0.00514	0.00617	0.04519
AC_AK_SF_A04_125	Alek	0.00760	0.00651	0.09528
AC_AK_SF_A04_126	Alek	0.00589	0.00750	0.08350
AC_AK_SF_A04_127	Alek	0.00597	0.00543	0.09528
AC_AK_SF_A04_128	Alek	0.00474	0.00526	0.06680
AC_AK_SF_A04_129	Alek	0.00358	0.00715	0.03831
AC_AK_SF_A04_130	Alek	0.00368	0.00526	0.06680
AC_AK_SF_A04_131	Alek	0.00530	0.00847	0.07269
AC_AK_SF_A04_132	Alek	0.00758	0.01299	0.09234
AC_AK_SF_A04_133	Alek	0.00315	0.00420	0.06483
AC_AK_SF_A04_134	Alek	0.00364	0.00520	0.05501
AC_AK_SF_A04_135	Alek	0.00514	0.00822	0.04420
AC_AK_SF_A04_136	Alek	0.00583	0.00954	0.07367
AC_AK_SF_LBF_176	Alek	0.00888	0.01523	0.22593
AC_AK_SF_LBF_177	Alek	0.00926	0.01058	0.25737
AC_AK_SF_LBF_178	Alek	0.00455	0.00607	0.35265
AC_AK_SF_LBF_179	Alek	0.00541	0.00812	0.27407

(B) Sample ID	Taxon	Hybrid index	Interclass heterozygosity	Proportion missing data
AC_AK_AF_A01_011a	Sa	0.0000	0.0000	0.1552
AC_AK_AF_A01_012	Sa	0.0000	0.0000	0.0992
AC_AK_AF_A01_013	Sa	0.0000	0.0000	0.1081
AC_AK_AF_A01_014b	Sa	0.0000	0.0000	0.0796
AC_AK_AF_A01_015c	Sa	0.0000	0.0000	0.4047
AC_AK_AF_A01_016d	Sa	0.0000	0.0000	0.0904
AC_AK_AF_A01_017	Sa	0.0000	0.0000	0.2122
AC_AK_AF_A02_018	Sa	0.0000	0.0000	0.1945
AC_AK_AF_A02_019	Sa	0.0000	0.0000	0.1876
AC_AK_AF_A02_020	Sa	0.0000	0.0000	0.3654
AC_AK_AF_A02_021	Sa	0.0000	0.0000	0.3880
AC_AK_AF_A04_022	Sa	0.0000	0.0000	0.2436
AC_AK_AF_A04_023	Sa	0.0000	0.0000	0.0992
AC_AK_AF_A04_024	Sa	0.0000	0.0000	0.0422
AC_AK_AF_A04_025	Sa	0.0000	0.0000	0.0796
AC_AK_AF_A04_026	Sa	0.0000	0.0000	0.0599
AC_AK_AF_A04_027	Sa	0.0000	0.0000	0.0678
AC_AK_AF_A04_028	Sa	0.0000	0.0000	0.0904
AC_AK_AF_A04_029	Sa	0.0000	0.0000	0.0619
AC_AK_AF_A04_030	Sa	0.0000	0.0000	0.0481
AC_AK_AF_A04_031	Sa	0.0000	0.0000	0.0786
AC_AK_AF_A04_032	Sa	0.0000	0.0000	0.0864
AC_AK_AF_A04_033	Sa	0.0000	0.0000	0.0756
AC_AK_AF_A04_034	Sa	0.0000	0.0000	0.0481
AC_AK_AF_A04_035	Sa	0.0000	0.0000	0.3222
AC_AK_AF_A04_036	Sa	0.0000	0.0000	0.0619
AC_AK_AF_A04_037	Sa	0.0000	0.0000	0.0845
AC_AK_AF_A04_038	Sa	0.0000	0.0000	0.0855
AC_AK_AF_A04_039e	Sa	0.0000	0.0000	0.0815
AC_AK_AF_A04_040e	Sa	0.0000	0.0000	0.0953
AC_AK_AF_A04_041	Sa	0.0000	0.0000	0.0540
AC_AK_AF_A04_042	Sa	0.0000	0.0000	0.0629
AC_AK_AF_A04_043	Sa	0.0000	0.0000	0.0688
AC_AK_AF_A04_044	Sa	0.0000	0.0000	0.0776
AC_AK_AF_A04_045	Sa	0.0000	0.0000	0.1031
AC_AK_AF_A04_046	Sa	0.0000	0.0000	0.0855
AC_AK_AF_A04_047	Sa	0.0000	0.0000	0.0560
AC_AK_AF_A04_048	Sa	0.0000	0.0000	0.0658
AC_AK_AF_A04_049	Sa	0.0000	0.0000	0.0658
AC_AK_AF_A04_050	Sa	0.0000	0.0000	0.1513
AC_AK_AF_A04_051b	Sa	0.0000	0.0000	0.0540
AC_AK_AF_A04_052	Sa	0.0000	0.0000	0.0874
AC_AK_AF_A04_053	Sa	0.0000	0.0000	0.0580
AC_AK_AF_A04_054d	Sa	0.0000	0.0000	0.0904
AC_AK_AF_A04_055c	Sa	0.0000	0.0000	0.0580
AC_AK_AF_A04_056	Sa	0.0000	0.0000	0.0678
AC_AK_AF_A04_057	Sa	0.0000	0.0000	0.1022
AC_AK_AF_A04_058	Sa	0.0000	0.0000	0.0550
AC_AK_AF_A04_059	Sa	0.0000	0.0000	0.1621
AC_AK_AF_A04_060	Sa	0.0000	0.0000	0.1336
AC_AK_AF_A04_061	Sa	0.0000	0.0000	0.0511
AC_AK_AF_A04_062	Sa	0.0000	0.0000	0.2387
AC_AK_AF_A04_064	Sa	0.0000	0.0000	0.0452
AC_AK_AF_A04_065	Sa	0.0000	0.0000	0.1365
AC_AK_AF_A04_066	Sa	0.0000	0.0000	0.2367
AC_AK_AF_A04_068	Sa	0.0000	0.0000	0.0914
AC_AK_AF_A04_069	Sa	0.0000	0.0000	0.0874
AC_AK_AF_A04_070	Sa	0.0000	0.0000	0.0707
AC_AK_AF_A04_071	Sa	0.0000	0.0000	0.0815
AC_AK_AF_A04_072	Sa	0.0000	0.0000	0.1002
AC_AK_AF_A04_073	Sa	0.0000	0.0000	0.1984
AC_AK_AF_A04_074	Sa	0.0000	0.0000	0.1719
AC_AK_AF_A04_075	Sa	0.0000	0.0000	0.0521
AC_AK_AF_A04_076	Sa	0.0000	0.0000	0.0432
AC_AK_AF_A04_077	Sa	0.0000	0.0000	0.0511
AC_AK_AF_A04_078	Sa	0.0000	0.0000	0.0648
AC_AK_AF_A04_079	Sa	0.0000	0.0000	0.0884
AC_AK_AF_A04_081	Sa	0.0000	0.0000	0.3861
AC_AK_AF_CHI_088a	Sa	0.0000	0.0000	0.1218
AC_AK_AF_LBF_089	Sa	0.0000	0.0000	0.3507
AC_AK_AF_LBF_090	Sa	0.0000	0.0000	0.2407
AC_AK_AF_LBF_091	Sa	0.0000	0.0000	0.4921
AC_AK_AF_LBF_092	Sa	0.0000	0.0000	0.3733
AC_AK_AF_LBF_093	Sa	0.0000	0.0000	0.3536
AC_AK_AF_LBF_094	Sa	0.0000	0.0000	0.1306
AC_AK_AF_LBF_095	Sa	0.0000	0.0000	0.5432
AC_AK_AF_LBF_096	Sa	0.0000	0.0000	0.5786
NDV_AK_AF_A01_250	Smm	1.0000	0.0000	0.6837
NDV_AK_AF_A01_251	Smm	1.0000	0.0000	0.2868
NDV_AK_AF_A01_252	Smm	1.0000	0.0000	0.4126
NDV_AK_AF_A02_253	Smm	1.0000	0.0000	0.2112
NDV_AK_AF_A02_254	Smm	1.0000	0.0000	0.2318
NDV_AK_AF_A02_255	Smm	1.0000	0.0000	0.2269
NDV_AK_AF_A04_256	Smm	1.0000	0.0000	0.1886
NDV_AK_AF_A04_258	Smm	1.0000	0.0000	0.0432
NDV_AK_AF_A04_259	Smm	1.0000	0.0000	0.0540
NDV_AK_AF_A04_260	Smm	1.0000	0.0000	0.0452
NDV_AK_AF_A04_261	Smm	1.0000	0.0000	0.0639
NDV_AK_AF_A04_262	Smm	1.0000	0.0000	0.0963
NDV_AK_AF_A04_263	Smm	1.0000	0.0000	0.0747
NDV_AK_AF_A04_264	Smm	1.0000	0.0000	0.0511
NDV_AK_AF_A04_265	Smm	1.0000	0.0000	0.0580
NDV_AK_AF_A04_266	Smm	1.0000	0.0000	0.0992
NDV_AK_AF_A04_267	Smm	1.0000	0.0000	0.0972
NDV_AK_AF_A04_268	Smm	1.0000	0.0000	0.0442
NDV_AK_AF_A04_269	Smm	1.0000	0.0000	0.0295
NDV_AK_AS_A01_270	Smm	1.0000	0.0000	0.0462
NDV_AK_AS_A01_271h	Smm	1.0000	0.0000	0.3527
NDV_AK_AS_A02_272	Smm	1.0000	0.0000	0.2151
NDV_AK_AS_A02_273	Smm	1.0000	0.0000	0.2662
NDV_AK_AS_A04_274	Smm	1.0000	0.0000	0.1090
NDV_AK_AS_A04_275	Smm	1.0000	0.0000	0.1955
NDV_AK_AS_A04_276	Smm	1.0000	0.0000	0.2387
NDV_AK_AS_A04_277	Smm	1.0000	0.0000	0.2033
NDV_AK_AS_A04_279	Smm	1.0000	0.0000	0.2338
NDV_AK_AS_A04_280	Smm	1.0000	0.0000	0.1562
NDV_AK_AS_A04_281	Smm	1.0000	0.0000	0.2927
NDV_AK_AS_A04_282	Smm	1.0000	0.0000	0.0167
NDV_AK_AS_A04_283	Smm	1.0000	0.0000	0.0560
NDV_AK_AS_A04_284	Smm	1.0000	0.0000	0.0432
NDV_AK_AS_A04_287	Smm	1.0000	0.0000	0.0992
NDV_AK_AS_A04_288	Smm	1.0000	0.0000	0.0354
NDV_AK_AS_A04_289	Smm	1.0000	0.0000	0.0796
NDV_AK_AS_A04_290	Smm	1.0000	0.0000	0.0334
NDV_AK_AS_A04_291	Smm	1.0000	0.0000	0.1139
NDV_AK_AS_A04_292	Smm	1.0000	0.0000	0.0884
NDV_AK_AS_A04_293	Smm	1.0000	0.0000	0.0530
NDV_AK_AS_A04_294	Smm	1.0000	0.0000	0.0796
NDV_AK_AS_A04_295	Smm	1.0000	0.0000	0.0373
NDV_AK_AS_A04_296	Smm	1.0000	0.0000	0.0246
NDV_AK_AS_A04_297	Smm	1.0000	0.0000	0.0472
NDV_AK_AS_A04_299	Smm	1.0000	0.0000	0.0403
NDV_AK_AS_A04_302	Smm	1.0000	0.0000	0.0432
NDV_AK_AS_A04_304	Smm	1.0000	0.0000	0.0717
NDV_AK_AS_A04_305	Smm	1.0000	0.0000	0.0727
NDV_AK_AS_A04_307	Smm	1.0000	0.0000	0.0491
NDV_AK_SF_A01_309	ILL	0.9866	0.0243	0.2308
NDV_AK_SF_A01_310	ILL	0.9798	0.0217	0.6837
NDV_AK_SF_A01_311	ILL	0.9775	0.0322	0.3890
NDV_AK_SF_A02_312	ILL	0.9850	0.0200	0.6061
NDV_AK_SF_A02_314	ILL	0.9851	0.0297	0.6031
NDV_AK_SF_A04_315	ILL	0.9882	0.0136	0.2083
NDV_AK_SF_A04_316	ILL	0.9911	0.0179	0.1198
NDV_AK_SF_A04_317	ILL	0.9884	0.0202	0.3202
NDV_AK_SF_A04_318	ILL	0.9700	0.0579	0.1002
NDV_AK_SF_A04_319	ILL	0.9818	0.0300	0.0845
NDV_AK_SF_A04_320	ILL	0.9840	0.0238	0.0501
NDV_AK_SF_A04_321	ILL	0.9832	0.0336	0.0933
NDV_AK_SF_A04_322	ILL	0.9807	0.0322	0.0835
NDV_AK_SF_A04_323	ILL	0.9834	0.0287	0.1444
NDV_AK_SF_A04_324	ILL	0.9864	0.0251	0.0982
NDV_AK_SF_A04_325	ILL	0.9862	0.0255	0.0747
NDV_AK_SS_A01_327i	ILL	0.9905	0.0127	0.0697
NDV_AK_SS_A04_370	ILL	0.9874	0.0210	0.0639
NDV_AK_SS_A04_371	ILL	0.9835	0.0268	0.0481
NDV_AK_SS_A04_372	ILL	0.9886	0.0228	0.0501
NDV_AK_SS_A04_373	ILL	0.9839	0.0260	0.0540
NDV_AK_SS_A04_374	ILL	0.9777	0.0339	0.0737
NDV_AK_SS_A04_375	ILL	0.9791	0.0299	0.0138
NDV_AK_SS_A04_376	ILL	0.9789	0.0320	0.0472
NDV_AK_SS_A04_377	ILL	0.9871	0.0196	0.0462
NDV_AK_SS_A04_378	ILL	0.9839	0.0258	0.0845
NDV_AK_SS_A04_379	ILL	0.9843	0.0271	0.0933
NDV_AK_SS_A04_380	ILL	0.9833	0.0313	0.0589
NDV_AK_SS_A04_381	ILL	0.9816	0.0305	0.0658
NDV_AK_SS_A04_382	ILL	0.9789	0.0340	0.0472
NDV_AK_SS_A04_383	ILL	0.9826	0.0286	0.0383
NDV_AK_SS_A04_384	ILL	0.9824	0.0248	0.0491
NDV_AK_SS_A04_385	ILL	0.9814	0.0248	0.0491
NDV_AK_SS_A04_386	ILL	0.9719	0.0499	0.0550
NDV_AK_SS_A04_387	ILL	0.9235	0.1529	0.2613
NDV_AK_SS_A04_388	ILL	0.9845	0.0289	0.0472
NDV_AK_SS_A04_389	ILL	0.8568	0.2838	0.2731
NDV_AK_SS_A04_390	ILL	0.9873	0.0253	0.1071
NDV_AK_SS_A04_391	ILL	0.9833	0.0233	0.0305
NDV_AK_SS_A04_392	ILL	0.9869	0.0240	0.0599
NDV_AK_SS_A04_393	ILL	0.9851	0.0256	0.0776
NDV_AK_SS_A04_394	ILL	0.9863	0.0274	0.1031
NDV_AK_SS_A04_395	ILL	0.9851	0.0215	0.0413
NDV_AK_SS_A04_396	ILL	0.9814	0.0288	0.0776
NDV_AK_SS_A04_397	ILL	0.9759	0.0461	0.0835
NDV_AK_SS_A04_398	ILL	0.9849	0.0259	0.0884
NDV_AK_SS_A04_399	ILL	0.9778	0.0339	0.0717
AC_AK_SF_A02_109	ILL	0.0434	0.0868	0.2868
AC_AK_SF_A02_110	ILL	0.0032	0.0064	0.2289
AC_AK_SF_A04_137	ILL	0.0032	0.0043	0.0923
AC_AK_SF_A04_138	ILL	0.0011	0.0021	0.0648
AC_AK_SF_A04_139	ILL	0.0052	0.0042	0.0560
AC_AK_SF_A04_140	ILL	0.0051	0.0080	0.1415
AC_AK_SF_A04_141	ILL	0.0055	0.0109	0.1906
AC_AK_SF_A04_142	ILL	0.0467	0.0935	0.1591
AC_AK_SF_A04_143	ILL	0.0104	0.0125	0.0550
AC_AK_SF_A04_144	ILL	0.0010	0.0021	0.0432
AC_AK_SF_A04_145	ILL	0.0072	0.0041	0.0403
AC_AK_SF_A04_146	ILL	0.2384	0.4768	0.2377
AC_AK_SF_A04_147	ILL	0.0036	0.0073	0.0540
AC_AK_SF_A04_148	ILL	0.0071	0.0141	0.0953
AC_AK_SF_A04_149	ILL	0.0036	0.0071	0.0363
AC_AK_SF_A04_150	ILL	0.0037	0.0074	0.0678
AC_AK_SF_A04_151	ILL	0.0048	0.0095	0.0717
AC_AK_SF_A04_152	ILL	0.0011	0.0023	0.1316
AC_AK_SF_LBF_180e	ILL	0.0012	0.0024	0.1955
AC_AK_SF_LBF_181f	ILL	0.0061	0.0123	0.4401
AC_AK_SF_LBF_182g	ILL	0.0077	0.0052	0.2377
AC_AK_SF_LBF_183	ILL	0.0039	0.0026	0.2397

(C) Sample ID	Taxon	Hybrid index	Interclass heterozygosity	Proportion missing data
AC_AK_AF_A01_011a	Sa	0.0000	0.0000	0.1484
AC_AK_AF_A01_012	Sa	0.0000	0.0000	0.0877
AC_AK_AF_A01_013	Sa	0.0000	0.0000	0.0947
AC_AK_AF_A01_014b	Sa	0.0000	0.0000	0.0832
AC_AK_AF_A01_015c	Sa	0.0000	0.0000	0.4107
AC_AK_AF_A01_016d	Sa	0.0000	0.0000	0.0896
AC_AK_AF_A01_017	Sa	0.0000	0.0000	0.2022
AC_AK_AF_A02_018	Sa	0.0000	0.0000	0.1689
AC_AK_AF_A02_019	Sa	0.0000	0.0000	0.1727
AC_AK_AF_A02_020	Sa	0.0000	0.0000	0.3615
AC_AK_AF_A02_021	Sa	0.0000	0.0000	0.3884
AC_AK_AF_A04_022	Sa	0.0000	0.0000	0.2527
AC_AK_AF_A04_023	Sa	0.0000	0.0000	0.0870
AC_AK_AF_A04_024	Sa	0.0000	0.0000	0.0358
AC_AK_AF_A04_025	Sa	0.0000	0.0000	0.0691
AC_AK_AF_A04_026	Sa	0.0000	0.0000	0.0531
AC_AK_AF_A04_027	Sa	0.0000	0.0000	0.0685
AC_AK_AF_A04_028	Sa	0.0000	0.0000	0.0813
AC_AK_AF_A04_029	Sa	0.0000	0.0000	0.0525
AC_AK_AF_A04_030	Sa	0.0000	0.0000	0.0422
AC_AK_AF_A04_031	Sa	0.0000	0.0000	0.0710
AC_AK_AF_A04_032	Sa	0.0000	0.0000	0.0755
AC_AK_AF_A04_033	Sa	0.0000	0.0000	0.0691
AC_AK_AF_A04_034	Sa	0.0000	0.0000	0.0448
AC_AK_AF_A04_035	Sa	0.0000	0.0000	0.3001
AC_AK_AF_A04_036	Sa	0.0000	0.0000	0.0576
AC_AK_AF_A04_037	Sa	0.0000	0.0000	0.0774
AC_AK_AF_A04_038	Sa	0.0000	0.0000	0.0838
AC_AK_AF_A04_039e	Sa	0.0000	0.0000	0.0729
AC_AK_AF_A04_040e	Sa	0.0000	0.0000	0.0851
AC_AK_AF_A04_041	Sa	0.0000	0.0000	0.0480
AC_AK_AF_A04_042	Sa	0.0000	0.0000	0.0557
AC_AK_AF_A04_043	Sa	0.0000	0.0000	0.0697
AC_AK_AF_A04_044	Sa	0.0000	0.0000	0.0742
AC_AK_AF_A04_045	Sa	0.0000	0.0000	0.1030
AC_AK_AF_A04_046	Sa	0.0000	0.0000	0.0768
AC_AK_AF_A04_047	Sa	0.0000	0.0000	0.0518
AC_AK_AF_A04_048	Sa	0.0000	0.0000	0.0646
AC_AK_AF_A04_049	Sa	0.0000	0.0000	0.0557
AC_AK_AF_A04_050	Sa	0.0000	0.0000	0.1292
AC_AK_AF_A04_051b	Sa	0.0000	0.0000	0.0550
AC_AK_AF_A04_052	Sa	0.0000	0.0000	0.0774
AC_AK_AF_A04_053	Sa	0.0000	0.0000	0.0518
AC_AK_AF_A04_054d	Sa	0.0000	0.0000	0.0845
AC_AK_AF_A04_055c	Sa	0.0000	0.0000	0.0480
AC_AK_AF_A04_056	Sa	0.0000	0.0000	0.0614
AC_AK_AF_A04_057	Sa	0.0000	0.0000	0.0985
AC_AK_AF_A04_058	Sa	0.0000	0.0000	0.0589
AC_AK_AF_A04_059	Sa	0.0000	0.0000	0.1478
AC_AK_AF_A04_060	Sa	0.0000	0.0000	0.1228
AC_AK_AF_A04_061	Sa	0.0000	0.0000	0.0473
AC_AK_AF_A04_062	Sa	0.0000	0.0000	0.2246
AC_AK_AF_A04_064	Sa	0.0000	0.0000	0.0441
AC_AK_AF_A04_065	Sa	0.0000	0.0000	0.1324
AC_AK_AF_A04_066	Sa	0.0000	0.0000	0.2156
AC_AK_AF_A04_068	Sa	0.0000	0.0000	0.0838
AC_AK_AF_A04_069	Sa	0.0000	0.0000	0.0729
AC_AK_AF_A04_070	Sa	0.0000	0.0000	0.0678
AC_AK_AF_A04_071	Sa	0.0000	0.0000	0.0781
AC_AK_AF_A04_072	Sa	0.0000	0.0000	0.0902
AC_AK_AF_A04_073	Sa	0.0000	0.0000	0.2060
AC_AK_AF_A04_074	Sa	0.0000	0.0000	0.1619
AC_AK_AF_A04_075	Sa	0.0000	0.0000	0.0480
AC_AK_AF_A04_076	Sa	0.0000	0.0000	0.0409
AC_AK_AF_A04_077	Sa	0.0000	0.0000	0.0461
AC_AK_AF_A04_078	Sa	0.0000	0.0000	0.0646
AC_AK_AF_A04_079	Sa	0.0000	0.0000	0.0877
AC_AK_AF_A04_081	Sa	0.0000	0.0000	0.3813
AC_AK_AF_CHI_088a	Sa	0.0000	0.0000	0.1260
AC_AK_AF_LBF_089	Sa	0.0000	0.0000	0.3314
AC_AK_AF_LBF_090	Sa	0.0000	0.0000	0.2278
AC_AK_AF_LBF_091	Sa	0.0000	0.0000	0.4658
AC_AK_AF_LBF_092	Sa	0.0000	0.0000	0.3538
AC_AK_AF_LBF_093	Sa	0.0000	0.0000	0.3295
AC_AK_AF_LBF_094	Sa	0.0000	0.0000	0.1228
AC_AK_AF_LBF_095	Sa	0.0000	0.0000	0.5502
AC_AK_AF_LBF_096	Sa	0.0000	0.0000	0.6008
NDV_AK_AF_A01_250	Smm	1.0000	0.0000	0.6942
NDV_AK_AF_A01_251	Smm	1.0000	0.0000	0.2764
NDV_AK_AF_A01_252	Smm	1.0000	0.0000	0.4044
NDV_AK_AF_A02_253	Smm	1.0000	0.0000	0.2009
NDV_AK_AF_A02_254	Smm	1.0000	0.0000	0.2194
NDV_AK_AF_A02_255	Smm	1.0000	0.0000	0.2239
NDV_AK_AF_A04_256	Smm	1.0000	0.0000	0.1747
NDV_AK_AF_A04_258	Smm	1.0000	0.0000	0.0422
NDV_AK_AF_A04_259	Smm	1.0000	0.0000	0.0531
NDV_AK_AF_A04_260	Smm	1.0000	0.0000	0.0422
NDV_AK_AF_A04_261	Smm	1.0000	0.0000	0.0627
NDV_AK_AF_A04_262	Smm	1.0000	0.0000	0.0825
NDV_AK_AF_A04_263	Smm	1.0000	0.0000	0.0717
NDV_AK_AF_A04_264	Smm	1.0000	0.0000	0.0499
NDV_AK_AF_A04_265	Smm	1.0000	0.0000	0.0589
NDV_AK_AF_A04_266	Smm	1.0000	0.0000	0.0979
NDV_AK_AF_A04_267	Smm	1.0000	0.0000	0.0870
NDV_AK_AF_A04_268	Smm	1.0000	0.0000	0.0448
NDV_AK_AF_A04_269	Smm	1.0000	0.0000	0.0313
NDV_AK_AS_A01_270	Smm	1.0000	0.0000	0.0429
NDV_AK_AS_A01_271h	Smm	1.0000	0.0000	0.3365
NDV_AK_AS_A02_272	Smm	1.0000	0.0000	0.2015
NDV_AK_AS_A02_273	Smm	1.0000	0.0000	0.2617
NDV_AK_AS_A04_274	Smm	1.0000	0.0000	0.1081
NDV_AK_AS_A04_275	Smm	1.0000	0.0000	0.1875
NDV_AK_AS_A04_276	Smm	1.0000	0.0000	0.2316
NDV_AK_AS_A04_277	Smm	1.0000	0.0000	0.1977
NDV_AK_AS_A04_279	Smm	1.0000	0.0000	0.2335
NDV_AK_AS_A04_280	Smm	1.0000	0.0000	0.1638
NDV_AK_AS_A04_281	Smm	1.0000	0.0000	0.2905
NDV_AK_AS_A04_282	Smm	1.0000	0.0000	0.0179
NDV_AK_AS_A04_283	Smm	1.0000	0.0000	0.0512
NDV_AK_AS_A04_284	Smm	1.0000	0.0000	0.0473
NDV_AK_AS_A04_287	Smm	1.0000	0.0000	0.0870
NDV_AK_AS_A04_288	Smm	1.0000	0.0000	0.0301
NDV_AK_AS_A04_289	Smm	1.0000	0.0000	0.0717
NDV_AK_AS_A04_290	Smm	1.0000	0.0000	0.0345
NDV_AK_AS_A04_291	Smm	1.0000	0.0000	0.1024
NDV_AK_AS_A04_292	Smm	1.0000	0.0000	0.0870
NDV_AK_AS_A04_293	Smm	1.0000	0.0000	0.0505
NDV_AK_AS_A04_294	Smm	1.0000	0.0000	0.0787
NDV_AK_AS_A04_295	Smm	1.0000	0.0000	0.0409
NDV_AK_AS_A04_296	Smm	1.0000	0.0000	0.0275
NDV_AK_AS_A04_297	Smm	1.0000	0.0000	0.0429
NDV_AK_AS_A04_299	Smm	1.0000	0.0000	0.0377
NDV_AK_AS_A04_302	Smm	1.0000	0.0000	0.0429
NDV_AK_AS_A04_304	Smm	1.0000	0.0000	0.0665
NDV_AK_AS_A04_305	Smm	1.0000	0.0000	0.0614
NDV_AK_AS_A04_307	Smm	1.0000	0.0000	0.0537
NDV_AK_SS_A01_332	Nerka	0.9887	0.0212	0.0633
NDV_AK_SS_A02_338	Nerka	0.9827	0.0346	0.1683
NDV_AK_SS_A02_339	Nerka	0.9899	0.0202	0.1145
NDV_AK_SS_A02_340	Nerka	0.9791	0.0404	0.1139
NDV_AK_SS_A04_400	Nerka	0.9711	0.0564	0.1152
NDV_AK_SS_A04_401	Nerka	0.9912	0.0148	0.0518
NDV_AK_SS_A04_402	Nerka	0.9874	0.0251	0.0825
NDV_AK_SS_A04_403	Nerka	0.9895	0.0183	0.0550
NDV_AK_SS_A04_404	Nerka	0.9892	0.0217	0.0544
NDV_AK_SS_A04_405	Nerka	0.9890	0.0206	0.0691
NDV_AK_SS_A04_406	Nerka	0.9829	0.0300	0.0838
NDV_AK_SS_A04_407	Nerka	0.9936	0.0115	0.0537
NDV_AK_SS_A04_408	Nerka	0.9871	0.0232	0.0358
NDV_AK_SS_A04_409	Nerka	0.9884	0.0231	0.0307
NDV_AK_SS_A04_410	Nerka	0.9928	0.0131	0.0717
NDV_AK_SS_A04_411	Nerka	0.7904	0.4174	0.2796
NDV_AK_SS_A04_412	Nerka	0.9920	0.0148	0.0461
NDV_AK_SS_A04_413	Nerka	0.9896	0.0167	0.0422
NDV_AK_SS_A04_414	Nerka	0.9852	0.0282	0.0461
NDV_AK_SS_A04_415	Nerka	0.9929	0.0142	0.0525
NDV_AK_SS_A04_416	Nerka	0.9890	0.0151	0.0653
NDV_AK_SS_A04_417	Nerka	0.9922	0.0129	0.0544
NDV_AK_SS_A04_418	Nerka	0.9893	0.0186	0.0736
NDV_AK_SS_A04_419	Nerka	0.9932	0.0124	0.0166
NDV_AK_SS_A04_420	Nerka	0.9936	0.0129	0.0576
NDV_AK_SS_A04_421	Nerka	0.9921	0.0158	0.0697
NDV_AK_SS_A04_422	Nerka	0.9916	0.0167	0.0429
NDV_AK_SS_LBF_461	Nerka	0.9911	0.0178	0.1715
NDV_AK_SS_LBF_462	Nerka	0.9885	0.0183	0.1631
NDV_AK_SS_LBF_463	Nerka	0.9847	0.0207	0.2258
NDV_AK_SS_LBF_464	Nerka	0.9795	0.0373	0.3135
NDV_AK_SS_LBF_465	Nerka	0.9860	0.0280	0.4971
AC_AK_SF_A01_104	Nerka	0.0088	0.0119	0.0883
AC_AK_SF_A01_105	Nerka	0.0075	0.0121	0.1030
AC_AK_SF_A01_106	Nerka	0.0046	0.0076	0.1612
AC_AK_SF_A02_111	Nerka	0.0081	0.0085	0.1676
AC_AK_SF_A02_112	Nerka	0.0069	0.0107	0.1599
AC_AK_SF_A02_113	Nerka	0.0076	0.0151	0.1542
AC_AK_SF_A02_114	Nerka	0.0053	0.0063	0.3954
AC_AK_SF_A02_115	Nerka	0.0069	0.0089	0.2131
AC_AK_SF_A02_116	Nerka	0.0174	0.0303	0.1555
AC_AK_SF_A04_153	Nerka	0.0276	0.0551	0.1408
AC_AK_SF_A04_154	Nerka	0.0045	0.0076	0.0697
AC_AK_SF_A04_155	Nerka	0.0025	0.0035	0.0915
AC_AK_SF_A04_156	Nerka	0.0074	0.0119	0.1369
AC_AK_SF_A04_157	Nerka	0.0037	0.0061	0.0505
AC_AK_SF_A04_158	Nerka	0.0953	0.1889	0.2175
AC_AK_SF_A04_159	Nerka	0.0070	0.0114	0.0422
AC_AK_SF_A04_160	Nerka	0.0092	0.0168	0.1267
AC_AK_SF_A04_161	Nerka	0.0074	0.0063	0.0902
AC_AK_SF_A04_162	Nerka	0.0058	0.0103	0.0678
AC_AK_SF_A04_163	Nerka	0.0076	0.0083	0.0742
AC_AK_SF_A04_164	Nerka	0.0937	0.1873	0.1836
AC_AK_SF_A04_165	Nerka	0.0052	0.0090	0.0806
AC_AK_SF_A04_166	Nerka	0.0057	0.0061	0.0505
AC_AK_SF_A04_167	Nerka	0.0147	0.0222	0.1062
AC_AK_SF_A04_168	Nerka	0.0054	0.0095	0.0544
AC_AK_SF_A04_169	Nerka	0.0143	0.0272	0.0819
AC_AK_SF_A04_170	Nerka	0.0039	0.0077	0.0902
AC_AK_SF_A04_171	Nerka	0.0060	0.0067	0.0422
AC_AK_SF_A04_172	Nerka	0.0055	0.0055	0.0627
AC_AK_SF_A04_173	Nerka	0.0097	0.0111	0.0755
AC_AK_SF_A04_174	Nerka	0.0067	0.0067	0.0473
AC_AK_SF_LBF_184	Nerka	0.0034	0.0068	0.1574
AC_AK_SF_LBF_185	Nerka	0.0041	0.0053	0.1491
AC_AK_SF_LBF_186	Nerka	0.0041	0.0049	0.2214
AC_AK_SF_LBF_187	Nerka	0.0063	0.0101	0.4933
AC_AK_SF_LBF_188	Nerka	0.2311	0.4545	0.6692

**TABLE A5:** Results of triangulaR analyses (Wiens et al. 2025) for allopatric *Salvelinus malma lordi* (Sml), allopatric *S. m*. *malma* (Smm) and admixed populations in Chignik Lake (A, Chignik), and Karluk Lake (B, Karluk), Alaska. Sample ID codes are defined in Table S1.

(A) Sample ID	Taxon	Hybrid index	Interclass heterozygosity	Proportion missing data
EPSDV_AK_AS_A02_488	Sml	1.0000	0.0000	0.2414
EPSDV_AK_AS_A04_489	Sml	1.0000	0.0000	0.0640
EPSDV_AK_AS_A04_490	Sml	1.0000	0.0000	0.0739
EPSDV_AK_AS_A04_491	Sml	1.0000	0.0000	0.0936
EPSDV_AK_AS_A04_492	Sml	1.0000	0.0000	0.1232
EPSDV_AK_AS_A04_493	Sml	1.0000	0.0000	0.0936
EPSDV_AK_AS_A04_494	Sml	1.0000	0.0000	0.1084
EPSDV_AK_AS_A04_495	Sml	1.0000	0.0000	0.0788
EPSDV_AK_AS_A04_496	Sml	1.0000	0.0000	0.0985
EPSDV_AK_AS_A04_497	Sml	1.0000	0.0000	0.1133
EPSDV_AK_NN_A04_498	Sml	1.0000	0.0000	0.1084
EPSDV_AK_NN_A04_499	Sml	1.0000	0.0000	0.0936
EPSDV_AK_NN_A04_500	Sml	1.0000	0.0000	0.0837
EPSDV_AK_NN_A04_501	Sml	1.0000	0.0000	0.1232
EPSDV_BC_AF_A01_502	Sml	1.0000	0.0000	0.3350
EPSDV_BC_AF_A01_503	Sml	1.0000	0.0000	0.1773
EPSDV_BC_AF_A01_504	Sml	1.0000	0.0000	0.0985
EPSDV_BC_AF_A01_505	Sml	1.0000	0.0000	0.1084
EPSDV_BC_AF_A01_506j	Sml	1.0000	0.0000	0.2266
EPSDV_BC_AF_A01_507	Sml	1.0000	0.0000	0.4680
EPSDV_BC_AF_A01_508	Sml	1.0000	0.0000	0.5025
EPSDV_BC_AF_A02_509	Sml	1.0000	0.0000	0.2315
EPSDV_BC_AF_A02_510	Sml	1.0000	0.0000	0.2365
EPSDV_BC_AF_A02_511	Sml	1.0000	0.0000	0.2020
EPSDV_BC_AF_A02_512	Sml	1.0000	0.0000	0.2956
EPSDV_BC_AF_A02_513	Sml	1.0000	0.0000	0.2709
EPSDV_BC_AF_CHI_514j	Sml	1.0000	0.0000	0.1626
EPSDV_BC_AF_LBF_516	Sml	1.0000	0.0000	0.5025
EPSDV_BC_AN_A04_517	Sml	1.0000	0.0000	0.1872
EPSDV_BC_AN_A04_518	Sml	1.0000	0.0000	0.1921
EPSDV_BC_AN_A04_519	Sml	1.0000	0.0000	0.1576
EPSDV_BC_AN_A04_520	Sml	1.0000	0.0000	0.0837
EPSDV_BC_AN_A04_521	Sml	1.0000	0.0000	0.1379
EPSDV_BC_AN_A04_522	Sml	1.0000	0.0000	0.2020
EPSDV_BC_AN_A04_523	Sml	1.0000	0.0000	0.0936
EPSDV_BC_AN_A04_524	Sml	1.0000	0.0000	0.2512
EPSDV_BC_AN_A04_525	Sml	1.0000	0.0000	0.0591
EPSDV_BC_AN_A04_526	Sml	1.0000	0.0000	0.1281
EPSDV_BC_AN_A04_527	Sml	1.0000	0.0000	0.1527
EPSDV_BC_AN_A04_528	Sml	1.0000	0.0000	0.2118
EPSDV_BC_AN_A04_529	Sml	1.0000	0.0000	0.2956
EPSDV_BC_AN_A04_530	Sml	1.0000	0.0000	0.1133
EPSDV_BC_AN_A04_531	Sml	1.0000	0.0000	0.1823
EPSDV_BC_AN_A04_532	Sml	1.0000	0.0000	0.0542
EPSDV_BC_AN_A04_533	Sml	1.0000	0.0000	0.2808
EPSDV_BC_AN_A04_534	Sml	1.0000	0.0000	0.2956
EPSDV_BC_AN_A04_535	Sml	1.0000	0.0000	0.0443
EPSDV_BC_AN_A04_536	Sml	1.0000	0.0000	0.1970
EPSDV_BC_AN_A04_537	Sml	1.0000	0.0000	0.0936
EPSDV_BC_AN_A04_538	Sml	1.0000	0.0000	0.1675
EPSDV_BC_AN_A04_539	Sml	1.0000	0.0000	0.0739
EPSDV_BC_AN_A04_540	Sml	1.0000	0.0000	0.0837
EPSDV_BC_AN_A04_541	Sml	1.0000	0.0000	0.1429
EPSDV_BC_AS_A01_542	Sml	1.0000	0.0000	0.1281
EPSDV_BC_AS_A01_543	Sml	1.0000	0.0000	0.3202
EPSDV_BC_AS_A01_544	Sml	1.0000	0.0000	0.1379
EPSDV_BC_AS_A01_545	Sml	1.0000	0.0000	0.1232
EPSDV_BC_AS_A01_546	Sml	1.0000	0.0000	0.1379
EPSDV_BC_AS_A01_547	Sml	1.0000	0.0000	0.1872
EPSDV_BC_AS_A01_548	Sml	1.0000	0.0000	0.1133
EPSDV_BC_AS_A01_549	Sml	1.0000	0.0000	0.0985
EPSDV_BC_AS_A02_550	Sml	1.0000	0.0000	0.3054
EPSDV_BC_AS_A02_551	Sml	1.0000	0.0000	0.2167
EPSDV_BC_AS_A02_552	Sml	1.0000	0.0000	0.2266
EPSDV_BC_AS_A02_553	Sml	1.0000	0.0000	0.5320
EPSDV_BC_AS_A02_554	Sml	1.0000	0.0000	0.2611
EPSDV_BC_AS_A02_555	Sml	1.0000	0.0000	0.2217
EPSDV_BC_AS_A02_556	Sml	1.0000	0.0000	0.6847
EPSDV_WA_AF_BWA_557	Sml	1.0000	0.0000	0.0640
EPSDV_WA_AF_BWA_558	Sml	1.0000	0.0000	0.1034
EPSDV_WA_AF_BWA_559	Sml	1.0000	0.0000	0.1084
EPSDV_WA_AF_BWA_560	Sml	1.0000	0.0000	0.1232
EPSDV_WA_AF_BWA_561	Sml	1.0000	0.0000	0.5271
EPSDV_WA_AF_BWA_562	Sml	1.0000	0.0000	0.0936
EPSDV_WA_AF_BWA_564	Sml	1.0000	0.0000	0.1281
EPSDV_WA_AF_BWA_565	Sml	1.0000	0.0000	0.0887
NDV_AK_AF_A01_250	Smm	0.0000	0.0000	0.5813
NDV_AK_AF_A01_251	Smm	0.0000	0.0000	0.2069
NDV_AK_AF_A01_252	Smm	0.0000	0.0000	0.4286
NDV_AK_AF_A02_253	Smm	0.0000	0.0000	0.2020
NDV_AK_AF_A02_254	Smm	0.0000	0.0000	0.2759
NDV_AK_AF_A02_255	Smm	0.0000	0.0000	0.2118
NDV_AK_AF_A04_258	Smm	0.0000	0.0000	0.0542
NDV_AK_AF_A04_259	Smm	0.0000	0.0000	0.0640
NDV_AK_AF_A04_260	Smm	0.0000	0.0000	0.0542
NDV_AK_AF_A04_261	Smm	0.0000	0.0000	0.0640
NDV_AK_AF_A04_262	Smm	0.0000	0.0000	0.0788
NDV_AK_AF_A04_263	Smm	0.0000	0.0000	0.0739
NDV_AK_AF_A04_264	Smm	0.0000	0.0000	0.0148
NDV_AK_AF_A04_265	Smm	0.0000	0.0000	0.0690
NDV_AK_AF_A04_266	Smm	0.0000	0.0000	0.0788
NDV_AK_AF_A04_267	Smm	0.0000	0.0000	0.0443
NDV_AK_AF_A04_268	Smm	0.0000	0.0000	0.0296
NDV_AK_AF_A04_269	Smm	0.0000	0.0000	0.0197
NDV_AK_AS_A01_271h	Smm	0.0000	0.0000	0.2857
NDV_AK_AS_A02_272	Smm	0.0000	0.0000	0.2315
NDV_AK_AS_A04_274	Smm	0.0000	0.0000	0.1182
NDV_AK_AS_A04_275	Smm	0.0000	0.0000	0.2266
NDV_AK_AS_A04_276	Smm	0.0000	0.0000	0.2167
NDV_AK_AS_A04_277	Smm	0.0000	0.0000	0.1626
NDV_AK_AS_A04_279	Smm	0.0000	0.0000	0.2020
NDV_AK_AS_A04_280	Smm	0.0000	0.0000	0.1379
NDV_AK_AS_A04_281	Smm	0.0000	0.0000	0.2315
NDV_AK_AS_A04_282	Smm	0.0000	0.0000	0.0246
NDV_AK_AS_A04_283	Smm	0.0000	0.0000	0.0443
NDV_AK_AS_A04_284	Smm	0.0000	0.0000	0.0690
NDV_AK_AS_A04_287	Smm	0.0000	0.0000	0.0985
NDV_AK_AS_A04_288	Smm	0.0000	0.0000	0.0345
NDV_AK_SF_A01_309	Smm	0.0000	0.0000	0.2217
NDV_AK_SF_A01_310	Smm	0.0000	0.0000	0.6059
NDV_AK_SF_A01_311	Smm	0.0000	0.0000	0.3793
NDV_AK_SF_A02_312	Smm	0.0000	0.0000	0.5764
NDV_AK_SF_A02_314	Smm	0.0000	0.0000	0.6108
NDV_AK_SF_A04_315	Smm	0.0000	0.0000	0.1970
NDV_AK_SF_A04_316	Smm	0.0000	0.0000	0.0739
NDV_AK_SF_A04_317	Smm	0.0000	0.0000	0.2956
NDV_AK_SF_A04_318	Smm	0.0000	0.0000	0.1576
NDV_AK_SF_A04_319	Smm	0.0000	0.0000	0.0443
NDV_AK_SF_A04_320	Smm	0.0000	0.0000	0.0443
NDV_AK_SF_A04_321	Smm	0.0000	0.0000	0.0640
NDV_AK_SF_A04_322	Smm	0.0000	0.0000	0.0837
NDV_AK_SF_A04_323	Smm	0.0000	0.0000	0.1379
NDV_AK_SF_A04_324	Smm	0.0000	0.0000	0.1626
NDV_AK_SF_A04_325	Smm	0.0000	0.0000	0.0493
NDV_AK_SS_A01_327i	Smm	0.0000	0.0000	0.0640
NDV_AK_SS_A01_328	Smm	0.0000	0.0000	0.6946
NDV_AK_SS_A01_330	Smm	0.0000	0.0000	0.0542
NDV_AK_SS_A01_331	Smm	0.0000	0.0000	0.0591
NDV_AK_SS_A01_332	Smm	0.0000	0.0000	0.0837
NDV_AK_SS_A02_335	Smm	0.0000	0.0000	0.1281
NDV_AK_SS_A02_336	Smm	0.0000	0.0000	0.1429
NDV_AK_SS_A02_337	Smm	0.0000	0.0000	0.0837
NDV_AK_SS_A02_338	Smm	0.0000	0.0000	0.1823
NDV_AK_SS_A02_339	Smm	0.0000	0.0000	0.1034
NDV_AK_SS_A02_340	Smm	0.0000	0.0000	0.0985
NDV_AK_SS_A04_344	Smm	0.0000	0.0000	0.5517
NDV_AK_SS_A04_345	Smm	0.0000	0.0000	0.6798
NDV_AK_SS_A04_347	Smm	0.0000	0.0000	0.6256
NDV_AK_SS_A04_348	Smm	0.0000	0.0000	0.5025
NDV_AK_SS_A04_349	Smm	0.0000	0.0000	0.4631
NDV_AK_SS_A04_350	Smm	0.0000	0.0000	0.0591
NDV_AK_SS_A04_351	Smm	0.0000	0.0000	0.0296
NDV_AK_SS_A04_352	Smm	0.0000	0.0000	0.1921
NDV_AK_SS_A04_353	Smm	0.0000	0.0000	0.0296
NDV_AK_SS_A04_354	Smm	0.0000	0.0000	0.0296
NDV_AK_SS_A04_355	Smm	0.0000	0.0000	0.0443
NDV_AK_SS_A04_356	Smm	0.0000	0.0000	0.0985
NDV_AK_SS_A04_357	Smm	0.0000	0.0000	0.1872
NDV_AK_SS_A04_358	Smm	0.0000	0.0000	0.0788
NDV_AK_SS_A04_359	Smm	0.0000	0.0000	0.0099
NDV_AK_SS_A04_360	Smm	0.0000	0.0000	0.0788
NDV_AK_SS_A04_362	Smm	0.0000	0.0000	0.0591
NDV_AK_SS_A04_363	Smm	0.0000	0.0000	0.0394
NDV_AK_SS_A04_364	Smm	0.0000	0.0000	0.0493
NDV_AK_SS_A04_365	Smm	0.0000	0.0000	0.0246
NDV_AK_SS_A04_366	Smm	0.0000	0.0000	0.0788
NDV_AK_SS_A04_367	Smm	0.0000	0.0000	0.0345
NDV_AK_SS_A04_368	Smm	0.0000	0.0000	0.0394
NDV_AK_SS_A04_369	Smm	0.0000	0.0000	0.0936
NDV_AK_SS_A04_370	Smm	0.0000	0.0000	0.0640
NDV_AK_SS_A04_371	Smm	0.0000	0.0000	0.0591
NDV_AK_SS_A04_372	Smm	0.0000	0.0000	0.0394
NDV_AK_SS_A04_373	Smm	0.0000	0.0000	0.0296
NDV_AK_SS_A04_374	Smm	0.0000	0.0000	0.0542
NDV_AK_SS_A04_375	Smm	0.0000	0.0000	0.0197
NDV_AK_SS_A04_376	Smm	0.0000	0.0000	0.0345
NDV_AK_SS_A04_377	Smm	0.0000	0.0000	0.0394
NDV_AK_SS_A04_378	Smm	0.0000	0.0000	0.0936
NDV_AK_SS_A04_379	Smm	0.0000	0.0000	0.0690
NDV_AK_SS_A04_380	Smm	0.0000	0.0000	0.0443
NDV_AK_SS_A04_381	Smm	0.0000	0.0000	0.0591
NDV_AK_SS_A04_382	Smm	0.0000	0.0000	0.0148
NDV_AK_SS_A04_383	Smm	0.0000	0.0000	0.0099
NDV_AK_SS_A04_384	Smm	0.0000	0.0000	0.0640
NDV_AK_SS_A04_385	Smm	0.0000	0.0000	0.0296
NDV_AK_SS_A04_387	Smm	0.0000	0.0000	0.1330
NDV_AK_SS_A04_388	Smm	0.0000	0.0000	0.0739
NDV_AK_SS_A04_389	Smm	0.0000	0.0000	0.1576
NDV_AK_SS_A04_390	Smm	0.0000	0.0000	0.0788
NDV_AK_SS_A04_391	Smm	0.0000	0.0000	0.0296
NDV_AK_SS_A04_392	Smm	0.0000	0.0000	0.0394
NDV_AK_SS_A04_393	Smm	0.0000	0.0000	0.0542
NDV_AK_SS_A04_394	Smm	0.0000	0.0000	0.0542
NDV_AK_SS_A04_395	Smm	0.0000	0.0000	0.0394
NDV_AK_SS_A04_396	Smm	0.0000	0.0000	0.0690
NDV_AK_SS_A04_397	Smm	0.0000	0.0000	0.0690
NDV_AK_SS_A04_398	Smm	0.0000	0.0000	0.0591
NDV_AK_SS_A04_399	Smm	0.0000	0.0000	0.0640
NDV_AK_SS_A04_400	Smm	0.0000	0.0000	0.0394
NDV_AK_SS_A04_401	Smm	0.0000	0.0000	0.0591
NDV_AK_SS_A04_402	Smm	0.0000	0.0000	0.0690
NDV_AK_SS_A04_403	Smm	0.0000	0.0000	0.0443
NDV_AK_SS_A04_404	Smm	0.0000	0.0000	0.0837
NDV_AK_SS_A04_405	Smm	0.0000	0.0000	0.0493
NDV_AK_SS_A04_406	Smm	0.0000	0.0000	0.0443
NDV_AK_SS_A04_407	Smm	0.0000	0.0000	0.0443
NDV_AK_SS_A04_408	Smm	0.0000	0.0000	0.0345
NDV_AK_SS_A04_409	Smm	0.0000	0.0000	0.0345
NDV_AK_SS_A04_410	Smm	0.0000	0.0000	0.0591
NDV_AK_SS_A04_412	Smm	0.0000	0.0000	0.0296
NDV_AK_SS_A04_413	Smm	0.0000	0.0000	0.0296
NDV_AK_SS_A04_414	Smm	0.0000	0.0000	0.0246
NDV_AK_SS_A04_415	Smm	0.0000	0.0000	0.0690
NDV_AK_SS_A04_416	Smm	0.0000	0.0000	0.0788
NDV_AK_SS_A04_417	Smm	0.0000	0.0000	0.0837
NDV_AK_SS_A04_418	Smm	0.0000	0.0000	0.0936
NDV_AK_SS_A04_419	Smm	0.0000	0.0000	0.0148
NDV_AK_SS_A04_420	Smm	0.0000	0.0000	0.0443
NDV_AK_SS_A04_421	Smm	0.0000	0.0000	0.0443
NDV_AK_SS_A04_422	Smm	0.0000	0.0000	0.0197
NDV_AK_SS_A04_423	Smm	0.0000	0.0000	0.1281
NDV_AK_SS_A04_424	Smm	0.0000	0.0000	0.3350
NDV_AK_SS_A04_425	Smm	0.0000	0.0000	0.4384
NDV_AK_SS_A04_426	Smm	0.0000	0.0000	0.0690
NDV_AK_SS_A04_427	Smm	0.0000	0.0000	0.1823
NDV_AK_SS_A04_428	Smm	0.0000	0.0000	0.0837
NDV_AK_SS_A04_429	Smm	0.0000	0.0000	0.2414
NDV_AK_SS_A04_430	Smm	0.0000	0.0000	0.0296
NDV_AK_SS_A04_431	Smm	0.0000	0.0000	0.0493
NDV_AK_SS_A04_432	Smm	0.0000	0.0000	0.0394
NDV_AK_SS_A04_433	Smm	0.0000	0.0000	0.2167
NDV_AK_SS_A04_434	Smm	0.0000	0.0000	0.0887
NDV_AK_SS_A04_435	Smm	0.0000	0.0000	0.3300
NDV_AK_SS_A04_436	Smm	0.0000	0.0000	0.0493
NDV_AK_SS_A04_437	Smm	0.0000	0.0000	0.0345
NDV_AK_SS_A04_438	Smm	0.0000	0.0000	0.0640
NDV_AK_SS_A04_439	Smm	0.0000	0.0000	0.1379
NDV_AK_SS_A04_440	Smm	0.0000	0.0000	0.1330
NDV_AK_SS_A04_441	Smm	0.0000	0.0000	0.0837
NDV_AK_SS_A04_442	Smm	0.0000	0.0000	0.0394
NDV_AK_SS_A04_443	Smm	0.0000	0.0000	0.0690
NDV_AK_SS_A04_444	Smm	0.0000	0.0000	0.1330
NDV_AK_SS_A04_445	Smm	0.0000	0.0000	0.1232
NDV_AK_SS_A04_446	Smm	0.0000	0.0000	0.0493
NDV_AK_SS_A04_447	Smm	0.0000	0.0000	0.1034
NDV_AK_SS_A04_448	Smm	0.0000	0.0000	0.0148
NDV_AK_SS_A04_449	Smm	0.0000	0.0000	0.0443
NDV_AK_SS_A04_450	Smm	0.0000	0.0000	0.0788
NDV_AK_SS_LBF_451	Smm	0.0000	0.0000	0.2759
NDV_AK_SS_LBF_452	Smm	0.0000	0.0000	0.2167
NDV_AK_SS_LBF_453	Smm	0.0000	0.0000	0.2857
NDV_AK_SS_LBF_454	Smm	0.0000	0.0000	0.3448
NDV_AK_SS_LBF_455	Smm	0.0000	0.0000	0.3498
NDV_AK_SS_LBF_456	Smm	0.0000	0.0000	0.5862
NDV_AK_SS_LBF_457i	Smm	0.0000	0.0000	0.4483
NDV_AK_SS_LBF_461	Smm	0.0000	0.0000	0.1823
NDV_AK_SS_LBF_462	Smm	0.0000	0.0000	0.1527
NDV_AK_SS_LBF_463	Smm	0.0000	0.0000	0.2709
NDV_AK_SS_LBF_464	Smm	0.0000	0.0000	0.3350
NDV_AK_SS_LBF_465	Smm	0.0000	0.0000	0.5172
NDV_AK_SS_LBF_466	Smm	0.0000	0.0000	0.6305
xNDVEPSDV_AK_AF_A01_635	Chignik	0.7405	0.3924	0.2217
xNDVEPSDV_AK_AF_A02_636	Chignik	0.4524	0.4167	0.1724
xNDVEPSDV_AK_AF_A02_637	Chignik	0.2326	0.3488	0.1527
xNDVEPSDV_AK_AF_CHI_638	Chignik	0.2982	0.3041	0.1576
xNDVEPSDV_AK_AF_CHI_639	Chignik	0.2972	0.4167	0.1133
xNDVEPSDV_AK_AF_CHI_640	Chignik	0.2787	0.3736	0.1429
xNDVEPSDV_AK_AF_CHI_641	Chignik	0.2923	0.3770	0.0985
xNDVEPSDV_AK_AF_CHI_642	Chignik	0.2923	0.3661	0.0985
xNDVEPSDV_AK_AF_CHI_643	Chignik	0.3895	0.4144	0.1084
xNDVEPSDV_AK_AF_CHI_644	Chignik	0.2814	0.3593	0.1773
xNDVEPSDV_AK_AF_CHI_645	Chignik	0.2529	0.3103	0.1429
xNDVEPSDV_AK_AF_CHI_646	Chignik	0.2816	0.2874	0.1429
xNDVEPSDV_AK_AF_CHI_647	Chignik	0.3613	0.4516	0.2365
xNDVEPSDV_AK_AF_CHI_648	Chignik	0.2203	0.2867	0.2956
xNDVEPSDV_AK_AF_CHI_649	Chignik	0.2781	0.3938	0.2118
xNDVEPSDV_AK_AF_CHI_650	Chignik	0.2647	0.3294	0.1626
xNDVEPSDV_AK_AF_CHI_651	Chignik	0.3174	0.4326	0.1232
xNDVEPSDV_AK_AF_CHI_652	Chignik	0.2861	0.3757	0.1478
xNDVEPSDV_AK_AF_CHI_653	Chignik	0.2447	0.2789	0.0640
xNDVEPSDV_AK_AF_CHI_654	Chignik	0.2939	0.3130	0.3547
xNDVEPSDV_AK_AF_CHI_655	Chignik	0.2908	0.3750	0.0936
xNDVEPSDV_AK_AF_CHI_656	Chignik	0.2887	0.4107	0.1724
xNDVEPSDV_AK_AF_CHI_657	Chignik	0.3056	0.3667	0.1133
xNDVEPSDV_AK_AF_CHI_658	Chignik	0.2974	0.3947	0.0640
xNDVEPSDV_AK_AF_CHI_659	Chignik	0.2694	0.4056	0.1133
xNDVEPSDV_AK_AF_CHI_660	Chignik	0.2882	0.3176	0.1626
xNDVEPSDV_AK_AF_CHI_661	Chignik	0.2933	0.3467	0.2611
xNDVEPSDV_AK_AF_CHI_662	Chignik	0.3237	0.3699	0.1478
xNDVEPSDV_AK_AF_CHI_663	Chignik	0.3147	0.3706	0.1626
xNDVEPSDV_AK_AF_CHI_664	Chignik	0.3017	0.3464	0.1182
xNDVEPSDV_AK_AF_CHI_665	Chignik	0.3125	0.3864	0.1330
xNDVEPSDV_AK_AF_CHI_666	Chignik	0.2571	0.3672	0.1281
xNDVEPSDV_AK_AF_CHI_667	Chignik	0.3143	0.4914	0.1379
xNDVEPSDV_AK_AF_CHI_668	Chignik	0.3434	0.4699	0.1823
xNDVEPSDV_AK_AF_CHI_669	Chignik	0.2799	0.3315	0.0936
xNDVEPSDV_AK_AF_CHI_670	Chignik	0.4564	0.4826	0.1527
xNDVEPSDV_AK_AF_CHI_671	Chignik	0.7154	0.3457	0.0739
xNDVEPSDV_AK_AF_CHI_672	Chignik	0.2486	0.3005	0.0985
xNDVEPSDV_AK_AF_CHI_673	Chignik	0.4628	0.5426	0.0739
xNDVEPSDV_AK_AF_CHI_674	Chignik	0.2233	0.3208	0.2167
xNDVEPSDV_AK_AF_CHI_675	Chignik	0.3064	0.4046	0.1478
xNDVEPSDV_AK_AF_CHI_676	Chignik	0.3182	0.3977	0.1330
xNDVEPSDV_AK_AF_CHI_677	Chignik	0.7824	0.3059	0.1626
xNDVEPSDV_AK_AF_CHI_678	Chignik	0.7690	0.2982	0.1576
xNDVEPSDV_AK_AF_CHI_679	Chignik	0.7529	0.3430	0.1527
xNDVEPSDV_AK_AF_CHI_680	Chignik	0.7823	0.2957	0.0837
xNDVEPSDV_AK_AF_CHI_681	Chignik	0.7716	0.2963	0.2020
xNDVEPSDV_AK_AF_CHI_682	Chignik	0.7431	0.2928	0.1084
xNDVEPSDV_AK_AF_CHI_683	Chignik	0.7316	0.2655	0.1281
xNDVEPSDV_AK_AF_CHI_684	Chignik	0.7799	0.2880	0.0936
xNDVEPSDV_AK_AF_CHI_685	Chignik	0.6594	0.3438	0.2118
xNDVEPSDV_AK_AF_CHI_686	Chignik	0.6503	0.3558	0.1970
xNDVEPSDV_AK_AF_CHI_687	Chignik	0.7458	0.2599	0.1281
xNDVEPSDV_AK_AF_CHI_688	Chignik	0.7355	0.3198	0.1527
xNDVEPSDV_AK_AF_CHI_689	Chignik	0.8195	0.2544	0.1675
xNDVEPSDV_AK_AF_CHI_690	Chignik	0.8063	0.3125	0.2118
xNDVEPSDV_AK_AF_CHI_691	Chignik	0.7119	0.3842	0.1281
xNDVEPSDV_AK_AF_CHI_692	Chignik	0.7910	0.3503	0.1281
xNDVEPSDV_AK_AF_CHI_693	Chignik	0.3047	0.4083	0.1675
xNDVEPSDV_AK_AF_CHI_694	Chignik	0.5090	0.4277	0.1823
xNDVEPSDV_AK_AF_CHI_695	Chignik	0.5145	0.4393	0.1478
xNDVEPSDV_AK_AF_CHI_696	Chignik	0.2260	0.2740	0.2808
xNDVEPSDV_AK_AF_CHI_697	Chignik	0.6594	0.4348	0.3202
xNDVEPSDV_AK_AF_CHI_698	Chignik	0.5276	0.4172	0.1970
xNDVEPSDV_AK_AF_CHI_699	Chignik	0.5212	0.4485	0.1872
xNDVEPSDV_AK_AF_CHI_700	Chignik	0.5989	0.4615	0.1034
xNDVEPSDV_AK_AF_CHI_701	Chignik	0.6842	0.3860	0.1576
xNDVEPSDV_AK_AF_CHI_702	Chignik	0.6734	0.3873	0.1478
xNDVEPSDV_AK_AF_CHI_703	Chignik	0.5629	0.3829	0.1379
xNDVEPSDV_AK_AF_CHI_704	Chignik	0.7708	0.3036	0.1724
xNDVEPSDV_AK_AF_CHI_705	Chignik	0.2778	0.3801	0.1576
xNDVEPSDV_AK_AF_CHI_706	Chignik	0.3758	0.4286	0.2069
xNDVEPSDV_AK_AF_CHI_707	Chignik	0.1889	0.2667	0.1133
xNDVEPSDV_AK_AF_CHI_708	Chignik	0.7950	0.2950	0.3153
xNDVEPSDV_AK_AF_CHI_709	Chignik	0.4082	0.3608	0.2217
xNDVEPSDV_AK_AF_CHI_710	Chignik	0.6436	0.4917	0.1084
xNDVEPSDV_AK_AF_CHI_711	Chignik	0.7331	0.4213	0.1232
xNDVEPSDV_AK_AF_CHI_712	Chignik	0.7719	0.3313	0.2118
xNDVEPSDV_AK_AF_CHI_713	Chignik	0.7692	0.2747	0.1034
xNDVEPSDV_AK_AF_CHI_714	Chignik	0.7159	0.3636	0.1330
xNDVEPSDV_AK_AF_CHI_715	Chignik	0.6994	0.3989	0.1232
xNDVEPSDV_AK_AF_CHI_716	Chignik	0.7606	0.3617	0.0739
xNDVEPSDV_AK_AF_CHI_717	Chignik	0.2472	0.3389	0.1133
xNDVEPSDV_AK_AF_CHI_718	Chignik	0.7096	0.4132	0.1773
xNDVEPSDV_AK_AF_CHI_719	Chignik	0.7098	0.3661	0.4483
xNDVEPSDV_AK_AF_CHI_720	Chignik	0.5240	0.4315	0.2808
xNDVEPSDV_AK_AF_CHI_721	Chignik	0.8074	0.2370	0.3350
xNDVEPSDV_AK_AF_CHI_722	Chignik	0.3462	0.4038	0.4877
xNDVEPSDV_AK_AF_CHI_723	Chignik	0.8382	0.2647	0.3300
xNDVEPSDV_AK_AF_CHI_724	Chignik	0.7640	0.3760	0.3842
xNDVEPSDV_AK_AF_CHI_725	Chignik	0.7081	0.4497	0.2660
xNDVEPSDV_AK_AF_CHI_727	Chignik	0.3109	0.3910	0.2315
xNDVEPSDV_AK_AF_CHI_728	Chignik	0.2241	0.3333	0.1429
xNDVEPSDV_AK_AF_CHI_729	Chignik	0.3621	0.3793	0.2857
xNDVEPSDV_AK_AF_CHI_730	Chignik	0.7171	0.3816	0.2512
xNDVEPSDV_AK_AF_CHI_731	Chignik	0.4491	0.5030	0.1773
xNDVEPSDV_AK_AF_CHI_732	Chignik	0.3980	0.4422	0.2759
xNDVEPSDV_AK_AF_CHI_733	Chignik	0.7089	0.2658	0.2217
xNDVEPSDV_AK_AF_CHI_734	Chignik	0.4545	0.3977	0.1330
xNDVEPSDV_AK_AF_CHI_735	Chignik	0.7697	0.3034	0.1232
xNDVEPSDV_AK_AF_CHI_736	Chignik	0.7695	0.3182	0.2414
xNDVEPSDV_AK_AF_CHI_737	Chignik	0.7341	0.3237	0.1478
xNDVEPSDV_AK_AF_CHI_738	Chignik	0.2143	0.2571	0.1379
xNDVEPSDV_AK_AF_CHI_739	Chignik	0.7707	0.3439	0.2266
xNDVEPSDV_AK_AF_CHI_740	Chignik	0.7755	0.2993	0.2759
xNDVEPSDV_AK_AF_CHI_741	Chignik	0.2258	0.2581	0.2365
xNDVEPSDV_AK_AF_CHI_742	Chignik	0.7357	0.3248	0.2266
xNDVEPSDV_AK_AF_CHI_743	Chignik	0.7184	0.3861	0.2217
xNDVEPSDV_AK_AF_CHI_744	Chignik	0.7604	0.2569	0.2906
xNDVEPSDV_AK_AF_CHI_745	Chignik	0.7452	0.3185	0.2266
xNDVEPSDV_AK_AF_CHI_746	Chignik	0.7202	0.5000	0.1724
xNDVEPSDV_AK_AF_CHI_747	Chignik	0.7568	0.3005	0.0985
xNDVEPSDV_AK_AF_CHI_748	Chignik	0.7827	0.2917	0.1724
xNDVEPSDV_AK_AF_CHI_749	Chignik	0.2579	0.3095	0.3793
xNDVEPSDV_AK_AF_CHI_750	Chignik	0.3097	0.3742	0.2365
xNDVEPSDV_AK_AF_CHI_751	Chignik	0.3446	0.3729	0.1281
xNDVEPSDV_AK_AF_CHI_752	Chignik	0.7313	0.3750	0.2118
xNDVEPSDV_AK_AF_CHI_753	Chignik	0.3800	0.5086	0.1379
xNDVEPSDV_AK_AF_CHI_754	Chignik	0.6604	0.4528	0.4778
xNDVEPSDV_AK_AF_CHI_755	Chignik	0.3092	0.3947	0.2512
xNDVEPSDV_AK_AF_CHI_756	Chignik	0.3994	0.5084	0.1182
xNDVEPSDV_AK_AF_CHI_757	Chignik	0.2213	0.2931	0.1429
xNDVEPSDV_AK_AF_CHI_758	Chignik	0.6092	0.4155	0.3005
xNDVEPSDV_AK_AF_CHI_759	Chignik	0.6413	0.4022	0.0936
xNDVEPSDV_AK_AF_CHI_760	Chignik	0.3832	0.4946	0.0936
xNDVEPSDV_AK_AF_CHI_761	Chignik	0.4179	0.4256	0.0394
xNDVEPSDV_AK_AF_CHI_762	Chignik	0.2869	0.3693	0.1330
xNDVEPSDV_AK_AF_CHI_763	Chignik	0.2904	0.3892	0.1773
xNDVEPSDV_AK_AF_CHI_764	Chignik	0.2275	0.3593	0.1773
xNDVEPSDV_AK_AF_CHI_765	Chignik	0.2514	0.3314	0.1379
xNDVEPSDV_AK_AF_CHI_766	Chignik	0.2784	0.3653	0.1773
xNDVEPSDV_AK_AF_CHI_767	Chignik	0.2440	0.3434	0.1823
xNDVEPSDV_AK_AF_CHI_768	Chignik	0.4352	0.3889	0.2020
xNDVEPSDV_AK_AF_CHI_769	Chignik	0.2974	0.3632	0.0640
xNDVEPSDV_AK_AF_CHI_770	Chignik	0.4274	0.5196	0.1182
xNDVEPSDV_AK_AF_CHI_771	Chignik	0.6878	0.3149	0.1084
xNDVEPSDV_AK_AF_CHI_772	Chignik	0.2620	0.3422	0.0788
xNDVEPSDV_AK_AF_CHI_773	Chignik	0.3005	0.3388	0.0985
xNDVEPSDV_AK_AF_CHI_774	Chignik	0.3686	0.4629	0.1379
xNDVEPSDV_AK_AF_CHI_775	Chignik	0.7367	0.3254	0.1675
xNDVEPSDV_AK_AF_CHI_776	Chignik	0.2767	0.3648	0.2167
xNDVEPSDV_AK_AF_CHI_777	Chignik	0.2669	0.3497	0.1970
xNDVEPSDV_AK_AF_CHI_778	Chignik	0.2865	0.3438	0.5271
xNDVEPSDV_AK_AF_CHI_780	Chignik	0.2925	0.2830	0.4778
xNDVEPSDV_AK_AF_CHI_781	Chignik	0.2704	0.2893	0.2167
xNDVEPSDV_AK_AF_CHI_782	Chignik	0.3080	0.3841	0.3202
xNDVEPSDV_AK_AF_CHI_783	Chignik	0.3671	0.4126	0.2956
xNDVEPSDV_AK_AF_CHI_784	Chignik	0.2243	0.2432	0.0887
xNDVEPSDV_AK_AF_CHI_785	Chignik	0.2385	0.3276	0.1429
xNDVEPSDV_AK_AF_CHI_786	Chignik	0.2516	0.3396	0.2167
xNDVEPSDV_AK_AF_CHI_787	Chignik	0.2904	0.3054	0.1773
xNDVEPSDV_AK_AF_CHI_788	Chignik	0.2861	0.4011	0.0788
xNDVEPSDV_AK_AF_CHI_789	Chignik	0.2903	0.3548	0.0837
xNDVEPSDV_AK_AF_CHI_790	Chignik	0.6826	0.3539	0.1232
xNDVEPSDV_AK_AF_CHI_791	Chignik	0.2678	0.3169	0.0985
xNDVEPSDV_AK_AF_CHI_792	Chignik	0.2899	0.4260	0.1675
xNDVEPSDV_AK_AF_CHI_793	Chignik	0.3036	0.3690	0.1724
xNDVEPSDV_AK_AF_CHI_794	Chignik	0.3703	0.4270	0.0887
xNDVEPSDV_AK_AF_CHI_795	Chignik	0.2406	0.3422	0.0788
xNDVEPSDV_AK_AF_CHI_796	Chignik	0.4886	0.5314	0.1379
xNDVEPSDV_AK_AF_CHI_797	Chignik	0.6366	0.4208	0.0985
xNDVEPSDV_AK_AF_CHI_798	Chignik	0.2532	0.3269	0.2315
xNDVEPSDV_AK_AF_CHI_799	Chignik	0.2933	0.3467	0.2611
xNDVEPSDV_AK_AF_CHI_800	Chignik	0.2456	0.3136	0.1675
xNDVEPSDV_AK_AF_CHI_801	Chignik	0.7562	0.3272	0.2020
xNDVEPSDV_AK_AF_CHI_802	Chignik	0.3886	0.4728	0.0936
xNDVEPSDV_AK_AF_CHI_803	Chignik	0.5042	0.5083	0.4089
xNDVEPSDV_AK_AF_CHI_804	Chignik	0.2725	0.3764	0.1232
xNDVEPSDV_AK_AF_CHI_805	Chignik	0.2178	0.3129	0.1970
xNDVEPSDV_AK_AF_CHI_806	Chignik	0.3730	0.3892	0.0887
xNDVEPSDV_AK_AF_CHI_807	Chignik	0.3918	0.4536	0.0443
xNDVEPSDV_AK_AF_CHI_808	Chignik	0.3315	0.3913	0.0936
xNDVEPSDV_AK_AF_CHI_809	Chignik	0.3827	0.4198	0.2020
xNDVEPSDV_AK_AF_CHI_810	Chignik	0.2967	0.4176	0.1034
xNDVEPSDV_AK_AF_CHI_811	Chignik	0.3159	0.4121	0.1034
xNDVEPSDV_AK_AF_CHI_812	Chignik	0.3583	0.4064	0.0788
xNDVEPSDV_AK_AF_CHI_813	Chignik	0.4148	0.3901	0.1034
xNDVEPSDV_AK_AF_CHI_814	Chignik	0.3931	0.4591	0.2167
xNDVEPSDV_AK_AF_CHI_815	Chignik	0.3158	0.4211	0.1576
xNDVEPSDV_AK_AF_CHI_816	Chignik	0.3611	0.4333	0.1133
xNDVEPSDV_AK_AF_CHI_817	Chignik	0.3735	0.3373	0.1823
xNDVEPSDV_AK_AF_CHI_818	Chignik	0.3257	0.4000	0.1379
xNDVEPSDV_AK_AF_CHI_819	Chignik	0.3343	0.3812	0.1084
xNDVEPSDV_AK_AF_CHI_820	Chignik	0.2409	0.2622	0.1921
xNDVEPSDV_AK_AF_CHI_821	Chignik	0.2554	0.3587	0.0936
xNDVEPSDV_AK_AF_CHI_822	Chignik	0.4045	0.3503	0.2266

(B) Sample ID	Taxon	Hybrid index	Interclass heterozygosity	Proportion missing data
EPSDV_BC_AF_A02_511	Sml	1.0000	0.0000	0.2020
EPSDV_BC_AF_A02_512	Sml	1.0000	0.0000	0.2956
EPSDV_BC_AF_A02_513	Sml	1.0000	0.0000	0.2709
EPSDV_BC_AF_CHI_514j	Sml	1.0000	0.0000	0.1626
EPSDV_BC_AF_LBF_516	Sml	1.0000	0.0000	0.5025
EPSDV_BC_AN_A04_517	Sml	1.0000	0.0000	0.1872
EPSDV_BC_AN_A04_518	Sml	1.0000	0.0000	0.1921
EPSDV_BC_AN_A04_519	Sml	1.0000	0.0000	0.1576
EPSDV_BC_AN_A04_520	Sml	1.0000	0.0000	0.0837
EPSDV_BC_AN_A04_521	Sml	1.0000	0.0000	0.1379
EPSDV_BC_AN_A04_522	Sml	1.0000	0.0000	0.2020
EPSDV_BC_AN_A04_523	Sml	1.0000	0.0000	0.0936
EPSDV_BC_AN_A04_524	Sml	1.0000	0.0000	0.2512
EPSDV_BC_AN_A04_525	Sml	1.0000	0.0000	0.0591
EPSDV_BC_AN_A04_526	Sml	1.0000	0.0000	0.1281
EPSDV_BC_AN_A04_527	Sml	1.0000	0.0000	0.1527
EPSDV_BC_AN_A04_528	Sml	1.0000	0.0000	0.2118
EPSDV_BC_AN_A04_529	Sml	1.0000	0.0000	0.2956
EPSDV_BC_AN_A04_530	Sml	1.0000	0.0000	0.1133
EPSDV_BC_AN_A04_531	Sml	1.0000	0.0000	0.1823
EPSDV_BC_AN_A04_532	Sml	1.0000	0.0000	0.0542
EPSDV_BC_AN_A04_533	Sml	1.0000	0.0000	0.2808
EPSDV_BC_AN_A04_534	Sml	1.0000	0.0000	0.2956
EPSDV_BC_AN_A04_535	Sml	1.0000	0.0000	0.0443
EPSDV_BC_AN_A04_536	Sml	1.0000	0.0000	0.1970
EPSDV_BC_AN_A04_537	Sml	1.0000	0.0000	0.0936
EPSDV_BC_AN_A04_538	Sml	1.0000	0.0000	0.1675
EPSDV_BC_AN_A04_539	Sml	1.0000	0.0000	0.0739
EPSDV_BC_AN_A04_540	Sml	1.0000	0.0000	0.0837
EPSDV_BC_AN_A04_541	Sml	1.0000	0.0000	0.1429
EPSDV_BC_AS_A01_542	Sml	1.0000	0.0000	0.1281
EPSDV_BC_AS_A01_543	Sml	1.0000	0.0000	0.3202
EPSDV_BC_AS_A01_544	Sml	1.0000	0.0000	0.1379
EPSDV_BC_AS_A01_545	Sml	1.0000	0.0000	0.1232
EPSDV_BC_AS_A01_546	Sml	1.0000	0.0000	0.1379
EPSDV_BC_AS_A01_547	Sml	1.0000	0.0000	0.1872
EPSDV_BC_AS_A01_548	Sml	1.0000	0.0000	0.1133
EPSDV_BC_AS_A01_549	Sml	1.0000	0.0000	0.0985
EPSDV_BC_AS_A02_550	Sml	1.0000	0.0000	0.3054
EPSDV_BC_AS_A02_551	Sml	1.0000	0.0000	0.2167
EPSDV_BC_AS_A02_552	Sml	1.0000	0.0000	0.2266
EPSDV_BC_AS_A02_553	Sml	1.0000	0.0000	0.5320
EPSDV_BC_AS_A02_554	Sml	1.0000	0.0000	0.2611
EPSDV_BC_AS_A02_555	Sml	1.0000	0.0000	0.2217
EPSDV_BC_AS_A02_556	Sml	1.0000	0.0000	0.6847
EPSDV_WA_AF_BWA_557	Sml	1.0000	0.0000	0.0640
EPSDV_WA_AF_BWA_558	Sml	1.0000	0.0000	0.1034
EPSDV_WA_AF_BWA_559	Sml	1.0000	0.0000	0.1084
EPSDV_WA_AF_BWA_560	Sml	1.0000	0.0000	0.1232
EPSDV_WA_AF_BWA_561	Sml	1.0000	0.0000	0.5271
EPSDV_WA_AF_BWA_562	Sml	1.0000	0.0000	0.0936
EPSDV_WA_AF_BWA_564	Sml	1.0000	0.0000	0.1281
EPSDV_WA_AF_BWA_565	Sml	1.0000	0.0000	0.0887
NDV_AK_AF_A01_250	Smm	0.0000	0.0000	0.5813
NDV_AK_AF_A01_251	Smm	0.0000	0.0000	0.2069
NDV_AK_AF_A01_252	Smm	0.0000	0.0000	0.4286
NDV_AK_AF_A02_253	Smm	0.0000	0.0000	0.2020
NDV_AK_AF_A02_254	Smm	0.0000	0.0000	0.2759
NDV_AK_AF_A02_255	Smm	0.0000	0.0000	0.2118
NDV_AK_AF_A04_258	Smm	0.0000	0.0000	0.0542
NDV_AK_AF_A04_259	Smm	0.0000	0.0000	0.0640
NDV_AK_AF_A04_260	Smm	0.0000	0.0000	0.0542
NDV_AK_AF_A04_261	Smm	0.0000	0.0000	0.0640
NDV_AK_AF_A04_262	Smm	0.0000	0.0000	0.0788
NDV_AK_AF_A04_263	Smm	0.0000	0.0000	0.0739
NDV_AK_AF_A04_264	Smm	0.0000	0.0000	0.0148
NDV_AK_AF_A04_265	Smm	0.0000	0.0000	0.0690
NDV_AK_AF_A04_266	Smm	0.0000	0.0000	0.0788
NDV_AK_AF_A04_267	Smm	0.0000	0.0000	0.0443
NDV_AK_AF_A04_268	Smm	0.0000	0.0000	0.0296
NDV_AK_AF_A04_269	Smm	0.0000	0.0000	0.0197
NDV_AK_AS_A01_271h	Smm	0.0000	0.0000	0.2857
NDV_AK_AS_A02_272	Smm	0.0000	0.0000	0.2315
NDV_AK_AS_A04_274	Smm	0.0000	0.0000	0.1182
NDV_AK_AS_A04_275	Smm	0.0000	0.0000	0.2266
NDV_AK_AS_A04_276	Smm	0.0000	0.0000	0.2167
NDV_AK_AS_A04_277	Smm	0.0000	0.0000	0.1626
NDV_AK_AS_A04_279	Smm	0.0000	0.0000	0.2020
NDV_AK_AS_A04_280	Smm	0.0000	0.0000	0.1379
NDV_AK_AS_A04_281	Smm	0.0000	0.0000	0.2315
NDV_AK_AS_A04_282	Smm	0.0000	0.0000	0.0246
NDV_AK_AS_A04_283	Smm	0.0000	0.0000	0.0443
NDV_AK_AS_A04_284	Smm	0.0000	0.0000	0.0690
NDV_AK_AS_A04_287	Smm	0.0000	0.0000	0.0985
NDV_AK_AS_A04_288	Smm	0.0000	0.0000	0.0345
NDV_AK_SF_A01_309	Smm	0.0000	0.0000	0.2217
NDV_AK_SF_A01_310	Smm	0.0000	0.0000	0.6059
NDV_AK_SF_A01_311	Smm	0.0000	0.0000	0.3793
NDV_AK_SF_A02_312	Smm	0.0000	0.0000	0.5764
NDV_AK_SF_A02_314	Smm	0.0000	0.0000	0.6108
NDV_AK_SF_A04_315	Smm	0.0000	0.0000	0.1970
NDV_AK_SF_A04_316	Smm	0.0000	0.0000	0.0739
NDV_AK_SF_A04_317	Smm	0.0000	0.0000	0.2956
NDV_AK_SF_A04_318	Smm	0.0000	0.0000	0.1576
NDV_AK_SF_A04_319	Smm	0.0000	0.0000	0.0443
NDV_AK_SF_A04_320	Smm	0.0000	0.0000	0.0443
NDV_AK_SF_A04_321	Smm	0.0000	0.0000	0.0640
NDV_AK_SF_A04_322	Smm	0.0000	0.0000	0.0837
NDV_AK_SF_A04_323	Smm	0.0000	0.0000	0.1379
NDV_AK_SF_A04_324	Smm	0.0000	0.0000	0.1626
NDV_AK_SF_A04_325	Smm	0.0000	0.0000	0.0493
NDV_AK_SS_A01_327i	Smm	0.0000	0.0000	0.0640
NDV_AK_SS_A01_328	Smm	0.0000	0.0000	0.6946
NDV_AK_SS_A01_330	Smm	0.0000	0.0000	0.0542
NDV_AK_SS_A01_331	Smm	0.0000	0.0000	0.0591
NDV_AK_SS_A01_332	Smm	0.0000	0.0000	0.0837
NDV_AK_SS_A02_335	Smm	0.0000	0.0000	0.1281
NDV_AK_SS_A02_336	Smm	0.0000	0.0000	0.1429
NDV_AK_SS_A02_337	Smm	0.0000	0.0000	0.0837
NDV_AK_SS_A02_338	Smm	0.0000	0.0000	0.1823
NDV_AK_SS_A02_339	Smm	0.0000	0.0000	0.1034
NDV_AK_SS_A02_340	Smm	0.0000	0.0000	0.0985
NDV_AK_SS_A04_344	Smm	0.0000	0.0000	0.5517
NDV_AK_SS_A04_345	Smm	0.0000	0.0000	0.6798
NDV_AK_SS_A04_347	Smm	0.0000	0.0000	0.6256
NDV_AK_SS_A04_348	Smm	0.0000	0.0000	0.5025
NDV_AK_SS_A04_349	Smm	0.0000	0.0000	0.4631
NDV_AK_SS_A04_350	Smm	0.0000	0.0000	0.0591
NDV_AK_SS_A04_351	Smm	0.0000	0.0000	0.0296
NDV_AK_SS_A04_352	Smm	0.0000	0.0000	0.1921
NDV_AK_SS_A04_353	Smm	0.0000	0.0000	0.0296
NDV_AK_SS_A04_354	Smm	0.0000	0.0000	0.0296
NDV_AK_SS_A04_355	Smm	0.0000	0.0000	0.0443
NDV_AK_SS_A04_356	Smm	0.0000	0.0000	0.0985
NDV_AK_SS_A04_357	Smm	0.0000	0.0000	0.1872
NDV_AK_SS_A04_358	Smm	0.0000	0.0000	0.0788
NDV_AK_SS_A04_359	Smm	0.0000	0.0000	0.0099
NDV_AK_SS_A04_360	Smm	0.0000	0.0000	0.0788
NDV_AK_SS_A04_362	Smm	0.0000	0.0000	0.0591
NDV_AK_SS_A04_363	Smm	0.0000	0.0000	0.0394
NDV_AK_SS_A04_364	Smm	0.0000	0.0000	0.0493
NDV_AK_SS_A04_365	Smm	0.0000	0.0000	0.0246
NDV_AK_SS_A04_366	Smm	0.0000	0.0000	0.0788
NDV_AK_SS_A04_367	Smm	0.0000	0.0000	0.0345
NDV_AK_SS_A04_368	Smm	0.0000	0.0000	0.0394
NDV_AK_SS_A04_369	Smm	0.0000	0.0000	0.0936
NDV_AK_SS_A04_370	Smm	0.0000	0.0000	0.0640
NDV_AK_SS_A04_371	Smm	0.0000	0.0000	0.0591
NDV_AK_SS_A04_372	Smm	0.0000	0.0000	0.0394
NDV_AK_SS_A04_373	Smm	0.0000	0.0000	0.0296
NDV_AK_SS_A04_374	Smm	0.0000	0.0000	0.0542
NDV_AK_SS_A04_375	Smm	0.0000	0.0000	0.0197
NDV_AK_SS_A04_376	Smm	0.0000	0.0000	0.0345
NDV_AK_SS_A04_377	Smm	0.0000	0.0000	0.0394
NDV_AK_SS_A04_378	Smm	0.0000	0.0000	0.0936
NDV_AK_SS_A04_379	Smm	0.0000	0.0000	0.0690
NDV_AK_SS_A04_380	Smm	0.0000	0.0000	0.0443
NDV_AK_SS_A04_381	Smm	0.0000	0.0000	0.0591
NDV_AK_SS_A04_382	Smm	0.0000	0.0000	0.0148
NDV_AK_SS_A04_383	Smm	0.0000	0.0000	0.0099
NDV_AK_SS_A04_384	Smm	0.0000	0.0000	0.0640
NDV_AK_SS_A04_385	Smm	0.0000	0.0000	0.0296
NDV_AK_SS_A04_387	Smm	0.0000	0.0000	0.1330
NDV_AK_SS_A04_388	Smm	0.0000	0.0000	0.0739
NDV_AK_SS_A04_389	Smm	0.0000	0.0000	0.1576
NDV_AK_SS_A04_390	Smm	0.0000	0.0000	0.0788
NDV_AK_SS_A04_391	Smm	0.0000	0.0000	0.0296
NDV_AK_SS_A04_392	Smm	0.0000	0.0000	0.0394
NDV_AK_SS_A04_393	Smm	0.0000	0.0000	0.0542
NDV_AK_SS_A04_394	Smm	0.0000	0.0000	0.0542
NDV_AK_SS_A04_395	Smm	0.0000	0.0000	0.0394
NDV_AK_SS_A04_396	Smm	0.0000	0.0000	0.0690
NDV_AK_SS_A04_397	Smm	0.0000	0.0000	0.0690
NDV_AK_SS_A04_398	Smm	0.0000	0.0000	0.0591
NDV_AK_SS_A04_399	Smm	0.0000	0.0000	0.0640
NDV_AK_SS_A04_400	Smm	0.0000	0.0000	0.0394
NDV_AK_SS_A04_401	Smm	0.0000	0.0000	0.0591
NDV_AK_SS_A04_402	Smm	0.0000	0.0000	0.0690
NDV_AK_SS_A04_403	Smm	0.0000	0.0000	0.0443
NDV_AK_SS_A04_404	Smm	0.0000	0.0000	0.0837
NDV_AK_SS_A04_405	Smm	0.0000	0.0000	0.0493
NDV_AK_SS_A04_406	Smm	0.0000	0.0000	0.0443
NDV_AK_SS_A04_407	Smm	0.0000	0.0000	0.0443
NDV_AK_SS_A04_408	Smm	0.0000	0.0000	0.0345
NDV_AK_SS_A04_409	Smm	0.0000	0.0000	0.0345
NDV_AK_SS_A04_410	Smm	0.0000	0.0000	0.0591
NDV_AK_SS_A04_412	Smm	0.0000	0.0000	0.0296
NDV_AK_SS_A04_413	Smm	0.0000	0.0000	0.0296
NDV_AK_SS_A04_414	Smm	0.0000	0.0000	0.0246
NDV_AK_SS_A04_415	Smm	0.0000	0.0000	0.0690
NDV_AK_SS_A04_416	Smm	0.0000	0.0000	0.0788
NDV_AK_SS_A04_417	Smm	0.0000	0.0000	0.0837
NDV_AK_SS_A04_418	Smm	0.0000	0.0000	0.0936
NDV_AK_SS_A04_419	Smm	0.0000	0.0000	0.0148
NDV_AK_SS_A04_420	Smm	0.0000	0.0000	0.0443
NDV_AK_SS_A04_421	Smm	0.0000	0.0000	0.0443
NDV_AK_SS_A04_422	Smm	0.0000	0.0000	0.0197
NDV_AK_SS_A04_423	Smm	0.0000	0.0000	0.1281
NDV_AK_SS_A04_424	Smm	0.0000	0.0000	0.3350
NDV_AK_SS_A04_425	Smm	0.0000	0.0000	0.4384
NDV_AK_SS_A04_426	Smm	0.0000	0.0000	0.0690
NDV_AK_SS_A04_427	Smm	0.0000	0.0000	0.1823
NDV_AK_SS_A04_428	Smm	0.0000	0.0000	0.0837
NDV_AK_SS_A04_429	Smm	0.0000	0.0000	0.2414
NDV_AK_SS_A04_430	Smm	0.0000	0.0000	0.0296
NDV_AK_SS_A04_431	Smm	0.0000	0.0000	0.0493
NDV_AK_SS_A04_432	Smm	0.0000	0.0000	0.0394
NDV_AK_SS_A04_433	Smm	0.0000	0.0000	0.2167
NDV_AK_SS_A04_434	Smm	0.0000	0.0000	0.0887
NDV_AK_SS_A04_435	Smm	0.0000	0.0000	0.3300
NDV_AK_SS_A04_436	Smm	0.0000	0.0000	0.0493
NDV_AK_SS_A04_437	Smm	0.0000	0.0000	0.0345
NDV_AK_SS_A04_438	Smm	0.0000	0.0000	0.0640
NDV_AK_SS_A04_439	Smm	0.0000	0.0000	0.1379
NDV_AK_SS_A04_440	Smm	0.0000	0.0000	0.1330
NDV_AK_SS_A04_441	Smm	0.0000	0.0000	0.0837
NDV_AK_SS_A04_442	Smm	0.0000	0.0000	0.0394
NDV_AK_SS_A04_443	Smm	0.0000	0.0000	0.0690
NDV_AK_SS_A04_444	Smm	0.0000	0.0000	0.1330
NDV_AK_SS_A04_445	Smm	0.0000	0.0000	0.1232
NDV_AK_SS_A04_446	Smm	0.0000	0.0000	0.0493
NDV_AK_SS_A04_447	Smm	0.0000	0.0000	0.1034
NDV_AK_SS_A04_448	Smm	0.0000	0.0000	0.0148
NDV_AK_SS_A04_449	Smm	0.0000	0.0000	0.0443
NDV_AK_SS_A04_450	Smm	0.0000	0.0000	0.0788
NDV_AK_SS_LBF_451	Smm	0.0000	0.0000	0.2759
NDV_AK_SS_LBF_452	Smm	0.0000	0.0000	0.2167
NDV_AK_SS_LBF_453	Smm	0.0000	0.0000	0.2857
NDV_AK_SS_LBF_454	Smm	0.0000	0.0000	0.3448
NDV_AK_SS_LBF_455	Smm	0.0000	0.0000	0.3498
NDV_AK_SS_LBF_456	Smm	0.0000	0.0000	0.5862
NDV_AK_SS_LBF_457i	Smm	0.0000	0.0000	0.4483
NDV_AK_SS_LBF_461	Smm	0.0000	0.0000	0.1823
NDV_AK_SS_LBF_462	Smm	0.0000	0.0000	0.1527
NDV_AK_SS_LBF_463	Smm	0.0000	0.0000	0.2709
NDV_AK_SS_LBF_464	Smm	0.0000	0.0000	0.3350
NDV_AK_SS_LBF_465	Smm	0.0000	0.0000	0.5172
NDV_AK_SS_LBF_466	Smm	0.0000	0.0000	0.6305
xNDVEPSDV_AK_SS_A01_858	Karluk	0.8556	0.2667	0.1133
xNDVEPSDV_AK_SS_A02_859	Karluk	0.9172	0.1065	0.1675
xNDVEPSDV_AK_SS_A02_860	Karluk	0.8618	0.2237	0.2512
xNDVEPSDV_AK_SS_A02_861	Karluk	0.8636	0.2208	0.2414
xNDVEPSDV_AK_SS_A02_862	Karluk	0.8647	0.2118	0.1626
xNDVEPSDV_AK_SS_A04_863	Karluk	0.7454	0.4601	0.1970
xNDVEPSDV_AK_SS_A04_864	Karluk	0.8231	0.2308	0.0394
xNDVEPSDV_AK_SS_A04_865	Karluk	0.8520	0.2179	0.1182
xNDVEPSDV_AK_SS_A04_866	Karluk	0.8353	0.2118	0.1626
xNDVEPSDV_AK_SS_A04_867	Karluk	0.8536	0.1934	0.1084
xNDVEPSDV_AK_SS_A04_868	Karluk	0.8629	0.2204	0.0837
xNDVEPSDV_AK_SS_A04_869	Karluk	0.8709	0.2033	0.1034
xNDVEPSDV_AK_SS_A04_870	Karluk	0.8542	0.1979	0.0542
xNDVEPSDV_AK_SS_A04_871	Karluk	0.9000	0.1474	0.0640
xNDVEPSDV_AK_SS_A04_872	Karluk	0.8785	0.1878	0.1084

**TABLE A6:** Results of triangulaR analyses (Wiens et al. 2025) for allopatric *S. m*. *malma* (Smm), allopatric *S. m. krascheninnikovi* (Smk) and admixed samples from the Kuril Islands (Kurils), Russia. Sample ID codes are defined in Table S1.

Sample ID	Taxon	Hybrid index	Interclass heterozygosity	Proportion missing data
NDV_RU_AN_A01_467	Smm	0.0000	0.0000	0.0835
NDV_RU_AN_A02_468	Smm	0.0000	0.0000	0.1976
NDV_RU_AN_A02_469	Smm	0.0000	0.0000	0.1627
NDV_RU_AN_A02_470	Smm	0.0000	0.0000	0.1474
NDV_RU_AN_A02_471	Smm	0.0000	0.0000	0.2121
SC_RU_AN_A01_482	Smm	0.0000	0.0000	0.0792
SC_RU_AN_A02_483	Smm	0.0000	0.0000	0.2112
SC_RU_AN_A04_487	Smm	0.0000	0.0000	0.4727
WPSDV_JP_AN_A01_566	Smk	1.0000	0.0000	0.1780
WPSDV_JP_AN_A01_567	Smk	1.0000	0.0000	0.1227
WPSDV_JP_AN_A01_568	Smk	1.0000	0.0000	0.0911
WPSDV_JP_AN_A02_569	Smk	1.0000	0.0000	0.2683
WPSDV_JP_AN_A02_570	Smk	1.0000	0.0000	0.1951
xNDVWPSDV_KUR_AN_A01_875	Smk	1.0000	0.0000	0.0809
xNDVWPSDV_KUR_AN_A04_890	Smk	1.0000	0.0000	0.0562
xNDVWPSDV_KUR_AN_A04_891	Smk	1.0000	0.0000	0.1320
xNDVWPSDV_KUR_AN_A04_876	Kurils	0.5517	0.8144	0.3160
xNDVWPSDV_KUR_AN_A04_877	Kurils	0.9555	0.0509	0.3305
xNDVWPSDV_KUR_AN_A04_881	Kurils	0.9494	0.0644	0.6295
xNDVWPSDV_KUR_AN_A04_883	Kurils	0.5363	0.7097	0.5775
xNDVWPSDV_KUR_AN_A04_884	Kurils	0.6889	0.3023	0.2223
xNDVWPSDV_KUR_AN_A04_885	Kurils	0.6832	0.3635	0.1235
xNDVWPSDV_KUR_AN_A04_886	Kurils	0.5005	0.9384	0.1559
xNDVWPSDV_KUR_AN_A04_887	Kurils	0.9480	0.0768	0.1244
xNDVWPSDV_KUR_AN_A04_888	Kurils	0.6065	0.3979	0.1244
xNDVWPSDV_KUR_AN_A04_889	Kurils	0.6362	0.3643	0.1559
xNDVWPSDV_KUR_AN_A04_892	Kurils	0.9455	0.0550	0.1482
xNDVWPSDV_KUR_AN_A04_893	Kurils	0.9495	0.0302	0.1814
